# Phages Preying on *Bacillus anthracis*, *Bacillus cereus*, and *Bacillus thuringiensis*: Past, Present and Future

**DOI:** 10.3390/v6072623

**Published:** 2014-07-09

**Authors:** Annika Gillis, Jacques Mahillon

**Affiliations:** Laboratory of Food and Environmental Microbiology, Université catholique de Louvain, Croix du Sud 2, L7.05.12, B-1348 Louvain-la-Neuve, Belgium

**Keywords:** (bacterio)phages, *Bacillus cereus* group, *Bacillus anthracis*, *Bacillus thuringiensis*, transducing phages, chromosomal prophages, plasmidial prophages, jumbo-phages, Gamma-like phages

## Abstract

Many bacteriophages (phages) have been widely studied due to their major role in virulence evolution of bacterial pathogens. However, less attention has been paid to phages preying on bacteria from the *Bacillus cereus* group and their contribution to the bacterial genetic pool has been disregarded. Therefore, this review brings together the main information for the *B. cereus* group phages, from their discovery to their modern biotechnological applications. A special focus is given to phages infecting *Bacillus anthracis*, *B. cereus* and *Bacillus thuringiensis*. These phages belong to the *Myoviridae*, *Siphoviridae*, *Podoviridae* and *Tectiviridae* families. For the sake of clarity, several phage categories have been made according to significant characteristics such as lifestyles and lysogenic states. The main categories comprise the transducing phages, phages with a chromosomal or plasmidial prophage state, γ-like phages and jumbo-phages. The current genomic characterization of some of these phages is also addressed throughout this work and some promising applications are discussed here.

## 1. Introduction

“A strong feeling of adventure is animating those who are working on bacterial viruses, a feeling that they have a small part in the great drive towards a fundamental problem in biology”.

—Max Delbrück, 1946 [1].

Although Delbrück’s avid expression forms part of his Harvey Lecture given almost 70 years ago, it is presently still being a trend, to invite those who might be interested to work on bacterial viruses to join a field that is wide open and full of possibilities. As the list of known bacterial viruses has grown ever since, so did our view of the global distribution of these entities. Currently, bacterial viruses are recognized as the most abundant biological entities on earth and can be found in all reservoirs populated by bacterial hosts [2]. With an estimated of more than 10^30^ tailed bacterial viruses in the biosphere [3], the effects of their infection in bacteria were certainly encountered by many bacteriologists prior their formal discovery, but it was not until early last century that bacterial viruses were reported and published twice, independently. The first report was done by Frederick W. Twort in 1915, who observed a lytic action on colonies of micrococci and succeeded in isolating the responsible agent [4]. Nevertheless, even though Twort mentioned in his famous note to *The Lancet*, that the observed phenomenon could be due to “an ultra-microscopic virus”, he only concluded that it was an infectious, filterable agent that killed bacteria and, in the process, multiplied itself [4,5]. Then, in 1917 Felix d’Herelle reported an agent lysing bacteria associated with dysentery [6]. D’Herelle named these infectious agents capable of lysing bacteria “bacteriophages” (shortened as phages) which literally means bacteria eaters (from *bacteria* and Greek φαγεῖν *phagein* “to eat”).

Shortly after their discovery, phages were used as therapeutic agents to control human pathogens [7]. However, after the emergence of the antibiotics, these agents received little attention and were kept almost in oblivion, except in countries where the access to antibiotics was difficult (e.g., Georgia as part of the former USSR). Notwithstanding this oversight though, during the 1940–1960s phages played a central role in the development of modern molecular biology. Nowadays, phages are getting back to the spotlight, not only for their molecular and biotechnological applications, but also as an alternative to combat multidrug resistance in bacteria due to their antibacterial and diagnostic (phage typing) properties. Besides, phages are recognized as molders of bacterial genome architecture, being able to act as potential reservoirs of “specialization” genes that then can be laterally transferred in different environments, providing the genetic adaptability of their host to its ecological niche [3,8,9].

Members of the *Bacillus cereus* group are known to be associated with many bacteriophages. This group of Gram-positive spore-forming bacteria, also referred as *B. cereus sensu lato* (*s.l.*), includes a range of versatile species of particular interest, mainly due to their capacity of causing human diseases and for their use in biotechnological applications [10,11]. The long history of research among members of this group has revealed their pathogenic potential and diverse host range. Actually, three members of the *B. cereus* group are well known, mostly due to their individual properties: *Bacillus anthracis*, the etiological agent of the lethal disease anthrax, *B. cereus sensu stricto* (herein referred to as *B. cereus*), the food contaminant and opportunistic human pathogen, and *Bacillus thuringiensis*, the insect pathogen used worldwide as bioinsecticide [12,13,14]. The highly specialized lifestyles displayed by these species are often directly associated with the acquisition of mobile genetic elements, particularly large plasmids, but also transposons, insertion sequences and phages.

In the *B. cereus* group, plasmids have come under close examination, especially those directly involved in pathogenicity, while phages have received less attention in terms of their potential contribution to the distinctive ecotypes and pathotypes. Nevertheless, approximately one decade ago, the interest in phages thriving in the *B. cereus* group revived, bringing to the table the question of whether and how these phages can contribute to the genetic diversity and niche adaptation observed in this lineage of bacteria. This issue is far from being solved, but some clues have arisen. Different studies have reflected what has been observed for phages infecting other bacterial species: the conversion of a bacterium from a non-pathogenic to a pathogenic existence is usually associated with the acquisition of virulence factors that can be mediated by phages [3]. Besides, it has been suggested that the gene pool of phages that infect the *B. cereus* group is large and diverse [15,16,17,18]. The aim of this review is to provide an overview of the main phages that have been described in this bacterial group from a genome-base perspective, with a special focus on phages preying on *B. anthracis*, *B. cereus* and *B. thuringiensis*. In addition, the potential of using these phages in medical, molecular and biotechnological applications is briefly discussed.

## 2. The *B. cereus* Group and Its Taxonomic Issues

According to the current taxonomy, the *B. cereus* group includes seven recognized species: *B. anthracis*, *B. cereus*, *B. thuringiensis*, *Bacillus weihenstephanensis*, *Bacillus mycoides*, *Bacillus pseudomycoides* and *Bacillus cytotoxicus*, which share a close genetic and biochemical kinship [19,20]. As it was previously mentioned, the three former members are mostly known because of their economical and clinical importance, having well-characterized phenotypical traits that have traditionally permitted to distinguish one from the other (Table 1), while the remaining members of the group are differentiated on the basis of major physiological (*i.e.*, psychrotolerance for *B. weihenstephanensis* and thermotolerance for *B. cytotoxicus*) and morphological (*i.e.*, rhizoidal growth in case of *B. mycoides* and *B. pseudomycoides*) characteristics [19,20,21].

**Table 1:** Characteristics commonly used to differentiate *B. anthracis*, *B. cereus* and *B. thuringiensis*.

Characteristics	*B. anthracis*	*B. cereus*	*B. thuringiensis*
Motility	No ^a^	Yes	Yes
Crystal parasporal inclusion(s)	No	No	Yes
Lysis by Gamma phage	Yes	No ^b^	No
Mucoid colony (capsule synthesis on bicarbonate medium)	Yes	No	No
Hemolytic activity on 5% blood agar (sheep or horse)	No ^a^	Yes ^c^	Yes ^c^
Penicillin resistance (β-lactamase production)	No ^a^	Yes	Yes
Phospholipase C activity	No	Yes ^c^	Yes ^c^
Chitinase activity	No	Yes	Yes
Tyrosine decomposition	No	Yes	Yes ^c^
Mutation non-sense in *plcR* regulator	Yes	No ^a^	No ^a^
Four genomic prophages	Yes	No	No

^a^ Occasional positive strains have been found. ^b^ Some atypical *B. cereus* strains can be infected by this phage. ^c^ Occasional negative strains have been found. Data extracted from [15,16,19,22,23].

The close relationship among the different members of the *B. cereus* group has been established by phylogenetic analyses of single or multiple gene markers and, recently, from data provided by multiple whole genome sequencing projects. Comparison of the 16S and 23S rRNA sequences of *B. cereus*, *B. anthracis* and *B. thuringiensis* revealed over 99% of identity [24,25,26,27]. Moreover, extensive genomic studies conducted on strains of *B. cereus*, *B. thuringiensis* and *B. anthracis* have suggested that it may be more appropriate to regard them as belonging to one generic species, *B. cereus s.l.*, from which various ecotypes and pathotypes have appeared [28,29,30,31]. The taxonomic problems of the *B. cereus* group have long been a source of confusion and discrepancy, as many of the species are genetically heterogeneous. Currently, seven major phylogenetic subdivisions can be distinguished among the members of *B. cereus s.l.*, with strains of *B. cereus*, *B. thuringiensis* and *B. anthracis* intermingled in these phylogenetic clusters [32,33,34]. To further complicate this matter, various mechanisms of horizontal gene transfer (*i.e.*, conjugation and/or transduction) are thought to have contributed to the emergence of the different ecotypes and pathotypes displayed by the *B. cereus* group, making the boundaries between the species blurred.

## 3. Pioneer Milestones

As already indicated, of all the members of the *B. cereus* group, *B. anthracis* and *B. thuringiensis* are probably the most important and well-studied species. Since the terrorist attacks in 2001 in the United States of America (USA), *B. anthracis* has revived for its use as a potential biological weapon. However, the anthrax disease has been known since ancient times and has always presented an occupational hazard to workers in agriculture, tanning, skinning, butchery, and bone crushing (for fertilizer). The anthrax bacillus was first described by Robert Koch in 1876, who demonstrated its reproductive cycle and that the spores, in the absence of bacteria, could cause anthrax. With this discovery, Koch became the first person to link a specific bacterium to a particular disease, although the germ theory of disease long preceded him [35].

Researchers have been looking for alternatives to control *B. anthracis* and may have come across phages and their lysing effects without knowing that they were viruses. For instance, in 1898, while studying *B. anthracis*, Nikolay Gamaleya discovered “bacteriolitic substances”, as it was later translated from Russian, that destroy microbes [36]. In the 1920s, several examples of “active principle”, “lytic principle”, “lytic filtrate” and, even, “pseudolytic reaction” were referring to filtrates that induced lysis of *B. anthracis* cells, most probably as a result of phages activity or lysis [37,38,39]. Furthermore, in 1929, a sort of phage therapy to treat anthrax disease was already proposed and experimented [40]. It was in 1930, however, that Cowles described a “lytic filtrate” that fulfilled the characteristics associated with the term “bacteriophage” [41]. This “phage” was isolated from crude sewage using *B. anthracis* Strasbourg strain and was active against 11 *B. anthracis* strains [41]. In the same study, Cowles stumbled upon two “atypical” *B. anthracis* strains that were resistant to the isolated phage. Thus, another bacteriophage active against both strains, but not against “typical” *B. anthracis*, was isolated from sewage [41]. As these two “atypical” strains were found to be motile, their identification as *B. anthracis* can be questionable (Table 1) and, presumably, they were *B. cereus* strains, possibly related to the group of strains known as “*B. cereus* variety *anthracis*” [42]. Moreover, with the discovery of host specificities of certain *B. anthracis* phages, the first phage-typing schemes for this bacterial group were assessed during the decade of 1950 [43]. In the early 1960s, the first electron micrographs showing the gross morphology of *B. anthracis* phages were obtained [44]. Ever since, several phages infecting *B. anthracis* have been isolated worldwide, including the well-known Gamma phage, that is currently used as a diagnostic tool to facilitate the distinction between *B. anthracis* and the other *B. cereus* group members (Table 1).

Despite the fact that *B. thuringiensis* was discovered in 1901, and rediscovered in 1911 [45]; the study of its phages occurred much later. The same phenomenon occurred for phages in *B. cereus.* One of the first reports of lysogeny in *B. cereus* that was associated with the presence of phage particles observed by electron microscopy was published in 1952 [46] and the first electron micrograph showing a phage associated with *B. thuringiensis* appeared in 1960 [47], but as for *B. anthracis* phages, these micrographs only showed the overall morphology of the phages. Since the early 1960s comparative and morphological studies of several phages of *B. thuringiensis* and *B. cereus* were performed [48,49]. As most studies during that decade focused on isolating phages from soil infecting, not only *B. thuringiensis,* but also *B. anthracis* and *B. cereus*, they were therefore designated the *cereus-anthracis-mycoides* group of phages or CAM [50,51]. The application of the negative contrast technique to electron microscopy considerably improved the examination of the fine structures of phages, and in particular those infecting the *B. cereus* group [52]. Several studies published during the late 1960s extended the view of the different morphologies displayed by phages infecting *B. thuringiensis*, along with their lifestyles [53,54,55]. In particular, Colasito and Rogoff isolated and characterized temperate and virulent phages of *B. thuringiensis* by assessing their morphology, host range, serum neutralization and adsorption rates, among other characteristics [54,55]. They were able to classify *B. thuringiensis* phages into morphological groups which are somewhat comparable with the current phage family classification (e.g., polyhedral head, contractile tail (equivalent to *Myoviridae*); polyhedral head, non-rigid straight tail (equivalent to *Siphoviridae*); oblong head, non-contractile straight short tail (equivalent to *Podoviridae*); see Section 4.1.) [54,55,56], emphasizing the importance of morphology as a key element for phage classification. Also, the book published by Tikhonenko in the late 1960s, where she studied through electron microscopy the fine structure of several phages of *B. mycoides* (e.g., phages No. 1, No. 19, N5, N17) and *B. anthracis*, among other phages from different genera, constitutes an invaluable reference today [57].

**Figure 1:**
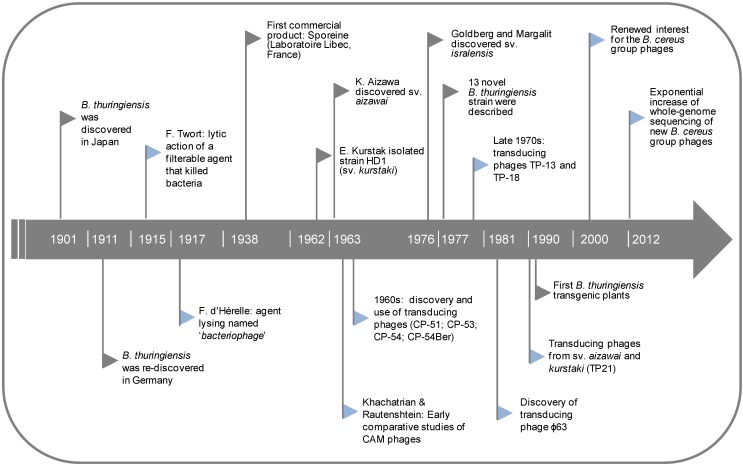
Timeline displaying the main milestones for *B. thuringiensis* research (grey flags) alongside the discovery of its phages (blue flags).

Furthermore, a DNA exchange system was found with the first generalized transducing phage, CP-51, isolated from *B. cereus* [58]. This transducing phage permitted the establishment of genomic maps in *B. thuringiensis* [59,60]. A noteworthy fact that characterizes the study of phages in *B. thuringiensis* between the 1960s and 1980s is that it was driven by the analysis of the genetic determinants of proteins responsible for insecticidal activities and the possibility of engineering strains for biotechnological applications. As can be seen in the timeline of Figure 1, the main phage reports for *B. thuringiensis* appeared side by side with milestones of *B. thuringiensis* research.

## 4. Phages of *B. anthracis*, *B. cereus* and *B. thuringiensis*

### 4.1. Classification of B. cereus Group Phages

Phages preying on the *B. cereus* group are of the double-stranded DNA (dsDNA) type and belong either to the order *Caudovirales* or the family *Tectiviridae*. So far, there are no reports for filamentous phages, single-stranded DNA or RNA phages in this bacterial group [56]. The order *Caudovirales*, featuring the tailed phages, is well represented by phages of the *B. cereus* group. In general, the morphology of the *Caudovirales* tail provides the basis for their classification into three families: *Myoviridae* (long contractile tails), *Siphoviridae* (long non-contractile tails) and *Podoviridae* (short non-contractile tails) (Table 2) [56]. The family *Tectiviridae* comprises icosahedral phages with an internal lipid vesicle that upon adsorption can act as a tail-like structure for genome delivery (Table 2, see Section 4.4.2.) [61]. The families *Myoviridae* and *Siphoviridae* are the most abundant in the *B. cereus* group phages.

**Table 2:** Characteristics of the phage families infecting *B. anthracis*, *B. cereus* and *B. thuringiensis*.

Order	Family	Morphology	Shape	Virion Size (nm)	Schematic Representation ^a^
*Caudovirales*	*Myoviridae*	Isometric head, contractile tail and a small base plate.	Tailed	Icosahedral heads: 50–145 Elongated heads: 80 × 110 Tail: 16–20 × 80–455	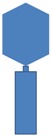
	*Siphoviridae*	Isometric head, long non-contractile tail. Some have elongated heads.	Tailed	Head: 40–80 Tail: 5–10 × 100–210	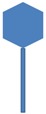
	*Podoviridae*	Isometric head, short non-contractile tail. Some have elongated heads.	Tailed	Head: 60–70 Tail: 10–20	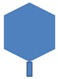
Unassigned	*Tectiviridae*	Isometric virion with apical spikes. Capsid encloses an inner membrane vesicle.	Polyhedral	Virion: 66 Spikes: 20	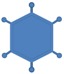

^a^ Not at scale. Data extracted from [56,61,62].

**Figure 2:**
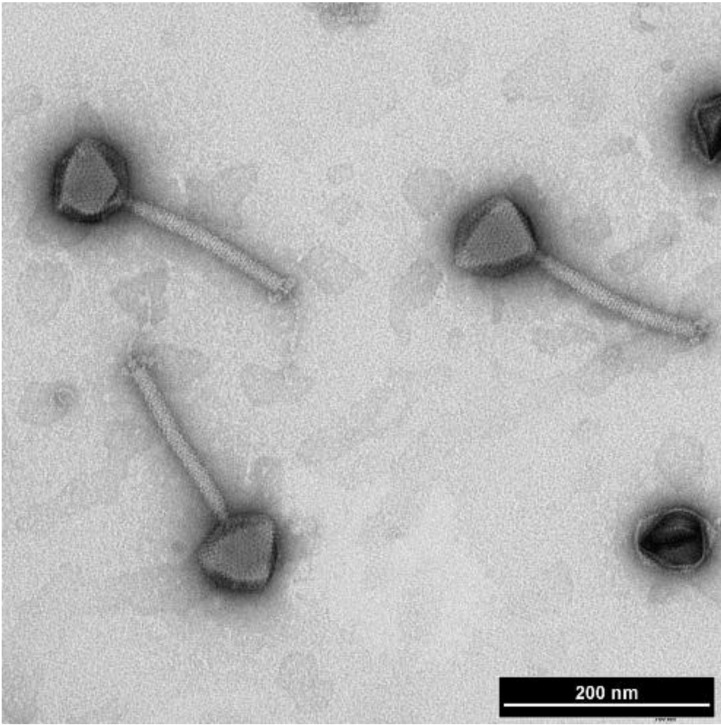
Transmission electron micrograph of the Twort-like phage vB_BceM_Bc431v3. Phage particles display isometric heads 85.4 ± 3 nm in diameter with individual capsomers. The phage possesses a long contractile tail 180 ± 3 nm in length by 12 ± 4 nm in width. Reproduced from El-Arabi *et al.* (2013), *Virol. J.* (reference [68]).

Interestingly, many myoviruses infecting the *B. cereus* group display characteristics typical of the recently proposed subfamily *Spounavirinae*. The *Spounavirinae* members have heads of about 84–94 nm in diameter and striated tails of 140–219 nm in length. The tail has globular structures at its tips, 6 short spikes and a double base plate. This subfamily includes the genera “SPO1-like viruses” and “Twort-like viruses” [63,64]. Recently however, phylogenetic analyses strongly suggest that the *Spounavirinae* are far more diverse than the current taxonomic arrangement [65]. The “SPO1-like” phages are large lytic phages with heads showing conspicuous capsomers. Additionally, their DNA is terminally redundant (but not circular permuted) and contains 5-hydroxymethyluracil (HMU) instead of thymine [56]. The “Twort-like” phages have longer tails (about 200 nm) and no HMU. This group is named after phage “Twort”, which may be a descendant of the original phage described in Twort’s article in 1915 [4,63]. Electron microscopy studies suggest that many of the *B. cereus* and *B. thuringiensis* myoviruses belong to the genus “Twort-like viruses” [64]. *B. cereus* phage vB_BceM_Bc431v3 (Figure 2; see Section 4.6.) is one of the phages with morphological characteristics similar to “Twort-like viruses”.

Despite the fact that phages preying on this group of bacteria are remarkably diverse from a “lifestyle and lysogenic state” point of view (e.g., virulent phages, phages integrated into the chromosome, integrated into plasmids or acting as independently replicating linear or circular elements) there is still a lack of information. Indeed, many interesting phages have not been sequenced yet, or were lost over time. Therefore, the information available for the non-sequenced *B. cereus* group phages referred in this review is collected in Table 3. Since many of the podoviruses found in the *B. cereus* group are poorly characterized, they are not included in this review, with the exception of *B. weihenstephanensis* phage MG-B1 (see Section 5.). Further information about some *B. cereus* group phages reported prior to 1985 that are not addressed in this review can be found in [66].

With the renewed interest for phages infecting the *B. cereus* group and the handiness of whole-genome sequencing technologies, numerous phage genomes have become available in the last years, greatly increasing our understanding of their genetic origin and diversity. All the fully-sequenced *B. cereus* group phages (to our knowledge, as of March 2014) are listed in Table 4 and, most of them, will be discussed throughout this review. While preparing this work, a comparative study of 30 sequenced *B. cereus s.l.* phages was performed by Lee and collaborators, revealing three genomic groups that correlate with some of the morphological phage families present in the *B. cereus* group (*i.e.*, *Myoviridae* for group I, *Siphoviridae* for group II, and *Tectiviridae* for group III) [67].

For the readily interpretation of this review, the available information has been divided in several phage categories that comprise fully-sequenced representatives, together with phages that feature remarkable characteristics, morphotypes, lifestyles and/or lysogenic states. The categories are as follows: transducing phages, phages with a chromosomal prophage state, γ-like phages, phages with a circular plasmidial prophage state, tectiviruses and jumbo-phages.

**Table 3:** Main features of *B. cereus*, *B. anthracis* and *B. thuringiensis* phages referred in this work whose DNA sequences have not been determined.

Morphology	Phage	(Original) Host	Estimated Genome Size	Lifestyle	Particular Features	References
*Myoviridae*	Bace-11	*B. cereus*	ND	ND	Virion morphology related to the 0305ϕ8-36 jumbo phage.	[69,70,71]
*Myoviridae*	BCP1-1	*B. cereus*	~150 kb	Virulent	Able to eradicate *B. cereus* from food. Divalent cations (Ca^2+^, Mg^2+^ or Mn^2+^) required for activity.	[72]
*Myoviridae*	BCP8-2	*B. cereus*	~150 kb	Virulent	Able to eradicate *B. cereus* from food Divalent cations (Ca^2+^, Mg^2+^ or Mn^2+^) required for activity.	[72]
*Myoviridae* (SPO1-like)	CP-51	*B. cereus* NRRL 569 (ATCC 10876)	~138 (88) kb ^a^	ND	Mediates generalized transduction. Instability at low temperatures. Infects sporulating *B. cereus* cells. Related to *B. subtilis* phage SPO1.	[58,59,64,73,74]
*Myoviridae*	CP-54	*B. thuringiensis* sv. *alesti* NRRL 4041	84–116 (339) kb ^a^	ND	Mediates generalized transduction.	[59]
*Myoviridae*	CP-54Ber	*B. thuringiensis* sv. *thuringiensis* Berliner 1715	84–116 (339) kb ^a^	ND	Mediates generalized transduction.	[75]
*Myoviridae*	FWLBc1	*B. cereus*	>90 kb	Virulent	Biocontrols *B. cereus*.	[76]
*Myoviridae*	FWLBc2	*B. cereus*	>90 kb	Virulent	Biocontrols *B. cereus*.	[76]
*Myoviridae*	JBP901	*B. cereus*	~150 kb	Virulent	Biocontrols *B. cereus* in liquid cultures and in fermented Korean food products.	[77]
*Myoviridae*	Tg13	*B. thuringiensis*	61 kb	ND	Mediates transduction.	[78]
*Myoviridae*	TP-13	*B. thuringiensis*	Possibly 380 kb	ND	Mediates generalized transduction. Converting phage for sporulation and crystal formation. Related to *B. subtilis* phages SP15.	[79]
*Myoviridae*	TP-18	*B. thuringiensis*	55 kb	ND	Mediates generalized transduction.	[60]
*Myoviridae*	Tt91	*B. thuringiensis*	ND	Virulent	Mediates specialized transduction.	[80]
*Siphoviridae*	ϕ20	*B. anthracis* Sterne 34F_2_ (pXO1^+^ pXO2^−^)	48.7 kb	ND	Has a circular plasmidial prophage state.	[81]
*Siphoviridae*	CP-53	*B. cereus* ATCC 6464	25 kb	ND	Mediates generalized transduction.	[73,74]
*Siphoviridae*	J7W-1	*B. thuringiensis* sv. *sotto/dendrolimus* AF101	48 kb	Temperate	Integrates into the 69 kb plasmid pAF101. Is induced by temperature or during mating.	[82,83,84]
*Siphoviridae*	MZTP01	*B. thuringiensis* sv. *kurstaki* MZ1	ND	Temperate	None.	[85]
*Siphoviridae*	Px1	*B. thuringiensis* sv. *galleriae* 69/6	ND	Temperate	Mediates transduction. Very sensitive to chloroform.	[86]
*Siphoviridae*	TP21-H	*B. thuringiensis*	ND	Temperate	None.	[87,88]
*Siphoviridae*	SU-11	*B. thuringiensis* sv. *israelensis*	ND	ND	Has a circular plasmidial prophage state.	[89]
*Tectiviridae*	Emet	*B. cereus* 5975c	~15 kb	Temperate	Isolated from an emetic *B. cereus*. Has a linear plasmidial prophage state.	[90]
*Tectiviridae*	Sand	*B. cereus* VD184	~15 kb	Temperate	Has a linear plasmidial prophage state.	[90]
*Tectiviridae*	Sato	*B. cereus* AND1284	~15 kb	Temperate	Isolated from an emetic *B. cereus*. Has a linear plasmidial prophage state.	[90]
*Tectiviridae*	Sole	*B. cereus* VD166	~15 kb	Temperate	Has a linear plasmidial prophage state.	[90]
ND	-	*B. anthracis*	ND	Virulent	First phage isolated for this bacterium.	[41]
ND	ϕ42	*B. thuringiensis* sv. *gelechiae* Bt1134	ND	ND	Mediates generalized transduction.	[91]
ND	ϕ63	*B. thuringiensis*	ND	ND	Mediates generalized transduction.	[91]
ND	ϕ64	*B. thuringiensis* sv. *alesti*	79–85 kb	ND	Mediates generalized transduction. Possibly a mutant of ϕ63.	[92]
ND	ϕHD67	*B. thuringiensis* sv. *alesti*	45.7 kb	Temperate	Mediates generalized transduction.	[93,94,95]
ND	ϕHD130	*B. thuringiensis*	38.1 kb	Temperate	Mediates transduction.	[93,94,95]
ND	ϕHD228	*B. thuringiensis*	36 kb	Temperate	Mediates transduction.	[93,94,95]
ND	ϕHD248	*B. thuringiensis*	47.1 kb	Temperate	Mediates transduction. Used for fine-structure chromosomal mapping.	[93,94,95]
ND	TP-21 (TP21-T)	*B. thuringiensis* sv. *kurstaki* HD-1 (HD1-9)	ND	ND	Mediates specialized transduction. Plasmidial phage.	[96,97]
ND	12826	*B. cereus* WS2453	ND	ND	Its endolysin (Ply12) lysis several *Bacillus* sp.	[98]
ND	BcpI	ND	ND	ND	Its endolysin (PlyB) has a potent lytic action against *B. anthracis*.	[99]
ND	TP-10	*B. thuringiensis*	ND	ND	Mediates generalized transduction.	[79]
ND	W	*B. cereus* W (ATCC 11950)	ND	Temperate	Parental phage for Gamma phage, probably same as Wβ.	[100]
ND	Wα	*B. cereus* W (ATCC 11950)	ND	Virulent	Rare virulent mutant of phage W.	[101]
ND	*wx*	*B. cereus* W (ATCC 11950)	ND	Temperate	Cryptic plasmid involved in lysogenic conversion to phospholipase A production.	[102,103]
ND	*wxc*	*B. cereus* W (ATCC 11950)	ND	Virulent	Virulent mutant of phage *wx*	[102]

^a^ See Table 5. ND: not determined.

**Table 4:** Genomic features of fully-sequenced *B. cereus* group phages ^a^.

Morphology ^b^	Phage	Host	Genome Size (bp)	GC%	Predicted ORFs	No. tRNAs	Lifestyle	GenBank Accession No.	Reference
*Myoviridae*	0305ϕ8-36	*B. thuringiensis*	218,948	41.80	247	2	Virulent	EF583821	[104]
*Myoviridae* (Twort-like)	B4	*B. cereus*	162,596	37.71	277	0	Virulent	JN790865	[105]
*Myoviridae* (Twort-like)	B5S	*B. cereus*	162,598	37.71	272	0	Virulent	JN797796	[67]
*Myoviridae* (Twort-like)	Bastille	*B. cereus*	153,962	38.14	273	7	Virulent	JF966203	[64]
*Myoviridae*	BCD7	*B. cereus*	93,839	38.04	140	0	Virulent	JN712910	-
*Myoviridae*	BCP78	*B. cereus*	156,176	39.86	227	18	Virulent	JN797797	[106]
*Myoviridae*	BCU4	*B. cereus*	154,371	39.86	223	19	Virulent	JN797798	[67]
*Myoviridae*	BigBertha	*B. thuringiensis*	162,661	37.80	287	0	Virulent ?	KF669647	[107]
*Myoviridae*	BPS10C	*B. cereus*	159,590	38.74	271	0	Virulent	KC430106	[108,109]
*Myoviridae*	BPS13	*B. cereus*	158,305	38.75	268	0	Virulent	JN654439	[108,109]
*Myoviridae*	JL	*B. cereus*	137,918	40.80	22	4	ND	KC595512	[110]
*Myoviridae*	Shanette	*B. cereus*	138,877	40.80	223	3	ND	KC595513	[110]
*Myoviridae*	Spock	*B. thuringiensis*	161,497	38.20	280	0	Virulent ?	KF669662	[111]
*Myoviridae*	Troll	*B. thuringiensis*	163,019	37.80	289	0	ND	KF208639	[112]
*Myoviridae* (Twort-like)	vB_BceM_Bc431v3	*B. cereus*	158,621	39.98	239	20	Virulent	JX094431	[68]
*Myoviridae*	W.Ph.	*B. cereus*	156,897	36.45	274	0	Virulent	HM144387	-
*Siphoviridae*	11143	*B. cereus*	39,077	34.96	49	0	Temperate	GU233956	[113]
*Siphoviridae*	250	*B. cereus*	56,505	36.45	54	0	Temperate	GU229986	[114]
*Siphoviridae*	Basilisk	*B. cereus*	81,790	33.90	140	2	ND	KC595511	[110]
*Siphoviridae*	BceA1	*B. cereus*	42,932	35.66	63	0	Temperate	HE614282	[115]
*Siphoviridae*	BMBtp2	*B. thuringiensis*	36,932	37.79	53	0	Temperate	JX887877	[116]
*Siphoviridae*	BtCS33	*B. thuringiensis*	41,992	35.22	57	0	Temperate	JN191664	[117]
*Siphoviridae*	Cherry ^c^	*B. anthracis*	36,615	35.26	51	0	Virulent	DQ222851	[118]
*Siphoviridae*	Fah ^c^	*B. anthracis*	37,974	34.94	50	0	Virulent	DQ150593	[119]
*Siphoviridae*	Gamma USAMRIID ^c^	*B. anthracis*	37,253	35.22	53	0	Virulent	DQ222853	[118]
*Siphoviridae*	Gamma LSU ^c^	*B. anthracis*	38,067	35.63	50	0	Virulent	DQ222855	[118]
*Siphoviridae*	Gamma isolate d’Herelle ^c^	*B. anthracis*	37,373	35.12	53	0	Virulent	DQ289556	[120]
*Siphoviridae*	Gamma Porton ^c^	*B. anthracis*	36,083	35.10	ND	0	Virulent	DQ221100	-
*Siphoviridae*	MZTP02	*B. thuringiensis*	15,717	37.55	20	0	Temperate	AY894696	[121]
*Siphoviridae*	PBC1	*B. cereus*	41,164	41.68	50	0	Virulent	JQ619704	[122]
*Siphoviridae*	phIS3501	*B. thuringiensis*	44,401	34.86	53	1	Temperate	JQ062992	[123]
*Siphoviridae*	phiCM3	*B. thuringiensis*	38,772	35.46	56	0	Virulent	KF296718	[124]
*Siphoviridae*	TP21-L	*B. cereus*	37,456	37.80	56	0	Temperate	EU887664	[88,98]
*Siphoviridae*	vB_BanS-Tsamsa	*B. anthracis*	168,876	37.80	272	19	Temperate	KC481682	[125]
*Siphoviridae*	vB_BceS-IEBH	*B. cereus*	53,104	36.42	86	0	Temperate	EU874396	[126]
*Siphoviridae*	Wβ	*B. cereus*	40,867	35.26	53	0	Temperate	DQ289555	[120]
*Podoviridae*	MG-B1	*B. weihenstephanensis*	27,190	30.75	43	0	Virulent	KC685370	[127]
*Tectiviridae*	AP50 ^d^	*B. anthracis*	14,398	38.65	31	0	Temperate	EU408779	[128]
*Tectiviridae*	Bam35 ^d^	*B. thuringiensis*	14,935	39.72	32	0	Temperate	AY257527	[129]
*Tectiviridae*	GIL01	*B. thuringiensis*	14,931	39.73	30	0	Temperate	AJ536073	[130]
*Tectiviridae*	GIL16 ^d^	*B. thuringiensis*	14,844	40.07	31	0	Temperate	AY701338	[131]
*Tectiviridae*	Wip1	*B. anthracis*	14,319	36.84	27	0	Temperate	KF188458	[132]
ND	lambdaBa01	*B. anthracis*	50,482	35.3	ND	0	Temperate	AE016879	[16]
ND	lambdaBa02	*B. anthracis*	44,043	35.0	ND	0	Temperate	AE016879	[16]
ND	lambdaBa03	*B. anthracis*	16,759	35.0	ND	0	Temperate	AE016879	[16]
ND	lambdaBa04	*B. anthracis*	37,385	34.0	ND	0	Temperate	AE016879	[16]
ND	phBC6A51	*B. cereus*	61,395	37.69	75	0	Temperate	NC_004820	[17]
ND	phBC6A52	*B. cereus*	38,472	34.72	49	0	Temperate	NC_004821	[17]
ND	proCM3	*B. thuringiensis*	43,278	37.40	58	0	Temperate	KF296717	[124]

^a^ *B. cereus* group phages available in GenBank as of March 2014. ^b^ Twort-like phages (subfamily *Spounavirinae*) are indicated. ^c^ Gamma phage isolate; lytic variant of phage Wβ. ^d^ Clear plaque mutant phage was used for genome sequencing. ND: not determined. ?: lifestyle not confirmed.

**Table 5:** Main characteristics of phages CP-51, CP-53, CP-54 and CP-54Ber.

Characteristics	CP-51	CP-53	CP-54	CP-54Ber
**Family**	*Myoviridae*	*Siphoviridae*	*Myoviridae*	*Myoviridae*
**Head diameter (nm)**	90	66	120–122	120
**Tail length (nm)**	160–185	276	198–200	200
**Estimated genome size (kb)**	138 (88) ^a^	25	84–116 (339) ^b^	84–116 (339) ^b^
**GC%**	43.9	37	43	43
**Genome particular features**	Fixed ends, HMU ^c^	No unusual bases	HMU	HMU
**Lifestyle**	Virulent	Temperate	Virulent	Temperate
**Transduction frequencies**	10^−7^–10^−5^	10^−7^–10^−6^	10^−7^–10^−5^	10^−7^–10^−5^
**Host range**	Active on different *B. thuringiensis*, *B.cereus* and *B. anthracis* strains	ND	Broader than CP-51	Narrower than CP-54, but active on *B.thuringiensis* sv. *thuringiensis* strain Berliner 1715
**Stability at 4 °C**	No	Yes	No (more cold sensitive than CP-51)	No
**Stability at 15 °C** **(plus divalent cations)**	Yes	ND	Yes (less than CP-51)	Yes (less than CP-51)
**Infection of sporulating cells**	Yes	ND	Yes	Yes

^a^ Discrepancy between measurements performed by [73] (88 kb) and [64] (138 kb). Since the last one was done by DNA sequencing, ~138 kb is assumed to be correct. ^b^ Discrepancy between measurements performed by [73,75] (84–116 kb) and [133] (339 kb). ^c^ 5-hydroxymethyluracil. ND: not determined. Data extracted from [58,64,73,74,75,133,134].

### 4.2. The Transducing Phages

Transduction is one of the modes of horizontal gene transfer in bacteria, by which some phages are able to mobilize bacterial genes from one bacterium to another. There are two types of transducing phages: *generalized* transducing phages that can carry any part of the chromosome and *specialized* transducing phages that carry only restricted parts of the bacterial chromosome [135]. Transduction, and more specifically generalized transduction, will be extensively addressed in this section, mainly because its important role in the construction of genomic and plasmid maps in this bacterial group.

#### 4.2.1. Phages CP-51, CP-53, CP-54 and CP-54Ber

Transduction experiments using phages of *B. anthracis*, *B. cereus* and *B. thuringiensis* were mainly done during the 1970s and used for genetic manipulations. As mentioned above, in 1968, Curtis Thorne isolated from soil the transducing phage **CP-51** using *B. cereus* NRRL 569 strain [58] (also known as *B. cereus* ATCC 10876). Later experiments showed that this phage was able to propagate on several other *B. cereus* strains (e.g., *B. cereus* ATCC 6464, ATCC 9139 and T) and also to mediate generalized transduction in some *B. anthracis* and *B. thuringiensis* strains [58,59,74,96,134]. During further transduction studies, Yelton and Thorne discovered a second phage, namely **CP-53**, in lysates of CP-51 propagated on *B. cereus* ATCC 6464, which mediated generalized transduction of *B. cereus* NRRL 569 auxotrophic mutants to prototrophy [74]. Interestingly, the authors indicated that CP-53 might be the same prophage that Altenbern and Stull found to be carried in *B. cereus* ATCC 6464 and was implicated in the increased release of edema factor and phospholipase [136,137], albeit, this has not been confirmed since. As presented in Table 5, phages CP-51 and CP-53 display different morphological features and transducing properties. One important characteristics of CP-51 is its instability at low temperatures, with 15 °C as the optimal temperature for maintenance in presence of divalent cations (Mg^+2^, Ca^+2^ or Mn^+2^) [58,134,138]. However, CP-51 exhibits greater co-transduction frequencies than CP-53 for linked markers, apparently due to its larger particle size which can carry more DNA than CP-53 [73]. To date, CP-51 is the most referenced transducing phage in the *B. cereus* group.

Remarkably, CP-51 was shown to mediate transfer of plasmid-encoded antibiotic resistances among several strains of *B. anthracis*, *B. cereus* and *B. thuringiensis* [96]. This ability was used to demonstrate that plasmid pXO2 encodes the genetic determinants necessary for the capsule synthesis in *B. anthracis* [139]. In these experiments, non-encapsulated *B. cereus* strains produced a capsule after CP-51-mediated transfer of pXO2. Moreover, it was demonstrated that CP-51 can infect sporulating *B. cereus* cells, in which phage DNA is trapped until spore germination [58]. Additional experiments revealed that CP-51 DNA transcription is suppressed at early stages of spore germination. After 45 min of germination, induction of phage RNA synthesis starts, occurring as a synchronous event and continuing at a similar rate as the one of the infected vegetative cells; cell lysis occurs at 100 min after initiation of phage development [140]. It was also shown that CP-51 is stable in infected spores of *B. thuringiensis* sv. *kurstaki* for at least 305 days even though most of the spores had lost refractility [141]. CP-51’s capability to infect sporulating cells was exploited to gain some insights into the response to spores nutrient germinants in *B. cereus* NRRL 569 [142]. Generalized transduction experiments using a heat-sensitive derivative of CP-51, named CP51ts, permitted to select transductants that confirmed the linkage of germination defects in *B. cereus* mutants to the resistance marker of the transposon used to generate those sporulation defective mutants; and, hence, to demonstrate that GerIA is present in *B. cereus* and it is involved in germination response to ribosides [142].

Further searches in soil samples for transducing phages on *B. thuringiensis* uncovered phage **CP-54**. This phage was isolated as described for CP-51, except that *B. thuringiensis* sv. *alesti* NRRL 4041 was used as host strain and streptomycin was omitted from the medium used [58,59]. Like CP-51, CP-54 is active on *B. cereus* NRRL 569. Therefore, this *B. cereus* strain was routinely used as indicator in CP-54 assays and infected spores as primary source of the phage. This myovirus possesses a tail with a neck of 10 × 8 nm, a sheath of 185 × 20 nm in the extended state and of 80 × 25 nm when contracted. It has a thin base plate and a system of about 40 nm long fibers with terminal clubs [133]. Although CP-54 and CP-51 are serologically related [59], their virion particles sizes are totally unlike. A first attempt to estimate CP-54 genome size indicated that it was between 84–116 kb [73,75]. However, it was shown later that CP-54 genome might be larger than previously thought (up to 339 kb) [133]. The initial (under)estimation of CP-54 genome size suggests that the phage DNA was broken during its extraction. The second CP-54 genome size estimation, alongside the phage head size, indicate that this phage might be a jumbo-phage or, even, a girus (see Section 4.5.).

A mutant of phage CP-54, denominated **CP-54Ber**, which was able to infect *B. thuringiensis* sv. *thuringiensis* strain Berliner 1715, was isolated after repeated subculturing of CP-54 lysates [75]. Phages CP-54 and CP-54Ber are similar in morphology, size and cryo-sensitivity. The main differences between them were shown to deal with inactivation by specific antiserum and host range. For both phages, co-transduction of genetic markers was demonstrated [59,75]. Later, CP-54Ber was used to transfer plasmid markers between several *B. thuringiensis* strains, a useful procedure for introducing crystal protein genes, responsible for the insecticidal activity, and thereby constructing novel strains with different gene combinations and biopesticidal activities [143]. As CP-54 and CP-54Ber morphologies are similar, CP-54Ber might be a jumbo-phage too, but their genome sequencing and further studies will confirm their classification.

Table 5 summarizes the main features that differentiate “CP-phages” (*i.e.*, CP-51, CP-53, CP-54 and CP-54Ber). It is worth noting that the cold-lability and the extremely virulent nature of phages CP-51 and CP-54 on some strains made them problematic to work with because the selection and scoring of transductants is difficult. However, with the use of some experimental modifications, like appropriate storing temperature, UV light to inactivate some phage particles and plating in enriched medium, reasonable yields of transductants are possible to obtain [59,138]. Additionally, some mutants of phages CP-51 (e.g., CP-51-26 and CP-51-4-59) and CP-54 (e.g., CP-54ant) with augmented transduction efficiencies have been obtained [144,145].

Hitherto, none of the genome sequences for the “CP-phages” have been released in public databases. Nevertheless, Klumpp and collaborators have found interesting characteristics when sequencing phage CP-51 genome [64]. The genome size was found to be approximately 138 kb with fixed (invariable) ends, coding for about 200 predicted ORFs and harboring two tRNAs. Because CP-51 shares 41% of similarity with genes that are present in *Bacillus subtilis* phage SPO1 (hit-length threshold 100 amino acid, 38%–100% identity), it was suggested that it belongs to the genus “SPO1-like viruses”, within the recently proposed subfamily *Spounavirinae* (see Section 4.1.) [63,64]. As most of the proposed SPO1-like viruses, the CP-51 genome contains HMU instead of thymine (Table 5) [63,64]. CP-51 genome also possesses fixed ends, a feature that does not correlate with its ability to transduce genetic markers [64]. It has been proposed that the observed infrequent transduction of CP-51 is due to occasional packing errors by the phage terminase holoenzyme [64]. Other attempts to complete the sequencing of phage CP-51 have been done using next generation sequencing (NGS) technologies, but are still facing some challenges such as PCR amplification biases and difficulties to sequence and assemble methylated bases [146].

#### 4.2.2. Phages TP-13 and TP-18

Following the isolation of the CP-phages, other phages that were able to mediate transduction among the *B. cereus* group have been identified, all with distinct transduction efficiencies. **TP-13** is a converting phage for sporulation and crystal formation in *B. thuringiensis* isolated from soil [79]. TP-13 was reported to be able to convert an oligo-sporogenic, acrystalliferous mutant to spore and crystal positive at a high frequency, and this conversion was shown to be independent of the host used for phage propagation. This phage is active on motile cells of at least 17 serovars of *B. thuringiensis* (except sv. *aizawai* NRRL 4048), and some strains of *B. cereus*. It mediates generalized transduction in several *B. thuringiensis* strains at frequencies of 10^−6^ to 10^−5^. TP-13 forms colony centered plaques on lawns of non-converted mutants, characteristic of plaques produced by temperate phages, but cells within the plaques do not sporulate [79]. Electron microscopy observations revealed that TP-13 belongs to the family *Myoviridae* and possess a head diameter of approximately 120 nm and a tail length of 260 nm, resembling the generalized transducing *B. subtilis* phage SP15 in morphology and size [79,147]. The head size of TP-13 is similar to that of SP15 and, thus, their genomes might be comparable [79] (SP15 estimated genome molecular mass by sedimentation coefficient technique: 250 MDa (ca. 380 kb) [147]). It was also shown that phages TP-13 and SP-15 are serologically related, but a common host has not been found [79].

Together with TP-13, another transducing phage, **TP-10**, was isolated from soil. Compared to TP-13 and CP-51, TP-10 is the smallest of the three and has the lowest cotransduction values [79]. Cotransduction value comparisons between phages CP-51 and TP-13 revealed that the latter transduces considerably larger segments of DNA [79], probably due to its larger head size. This characteristic, combined with the temperate nature of its plaques, made TP-13 an ideal candidate to be used in genome mapping studies. Actually, TP-13, in combination with phage **TP-18**, was successfully used for mapping genetic markers in *B. thuringiensis* [60]. Although TP-13 and TP-18 are morphologically related, TP-18 has a considerable smaller genome than TP-13, with an estimated genome molecular mass of 36 MDa (ca. 55 kb). Electron microscopic measurements of the head sizes suggested that the volume of TP-13 head is seven times greater than that of TP-18 (head diameter: 48 nm, head length: 89 nm, tail length: 175 nm). The small TP-18 genome size along with a smaller head size, compared to TP-13, might indicate also lower transduction rates than those of TP-13. TP-18 has a narrower host range than TP-13, being active only on nine out of 21 strains of *B. thuringiensis* tested. No motile cells are required for TP-18 infection. By means of TP-13 and TP-18, Barsomian and co-workers mapped three groups of linked markers in *B. thuringiensis* NRRL 4042B. While TP-13 was used to identify linkage groups since it packages relatively large pieces of DNA, TP-18 was used to determine the order of closely linked markers [60].

#### 4.2.3. Phages ϕ63 and its Derivative Mutant ϕ64

In order to have genetic exchanges systems to construct tailor-made insecticidal strains, another generalized transducing *B. thuringiensis* phage, **ϕ63**, was isolated from a soil sample by Landén and collaborators [91]. Using *B. thuringiensis* sv. *gelechiae* Bt1134, a second generalized transducing phage, **ϕ42**, was isolated alongside ϕ63 [91]. However, ϕ42 has lower transduction frequencies than ϕ63 and, therefore, it was not further characterized. ϕ63 forms turbid or clear plaques on 10 different serovars of *B. thuringiensis,* as well as on some strains of *B. cereus.* Remarkably, there was no plaque production on *B. thuringiensis* serovars *israelensis, aizawai* and *alesti*. Electron micrographs showed that ϕ63 morphology resembles that of TP-13, possessing a head diameter of 95 nm and a tail length of 200 nm. Landén and collaborators used ϕ63 to map the order of four antibiotic resistance genes (nalidixic acid, rifampicin, streptomycin and spectinomycin) of which the three last ones are part of a ribosomal cluster. The transduction frequencies regularly obtained for some of these markers were in the order of 10^−7^, an order of magnitude higher that when CP-54 was used to transduce the same markers [91]. Also, ϕ63 displays a greater stability and can be stored at 4 °C (stabilized with Ca^+2^) without the loss of titer that characterizes phages CP-51, CP-54 and CP-54Ber. ϕ63 is the first reported phage that could mediate cotransduction of more than two genes. In addition, it was demonstrated that ϕ63 transduces gene markers in five of the six serovars of *B. thuringiensis* tested [91].

What is believed to be a mutant of ϕ63, named **ϕ64**, was obtained during an attempt to purify and concentrate ϕ63 [92]. Host range and transduction abilities evaluations showed that these two phages differ in several aspects, despite that the inactivation curves using antiserum against ϕ63 gave identical profiles for both phages. Compared to ϕ63, ϕ64 was active on *B. thuringiensis* sv. *alesti* and its ability to transduce prototrophic markers (e.g., Leu^+^) was increased about 10 times. The estimated genome molecular mass for ϕ64 is around 52–56 MDa (79–85 kb). Phage ϕ64 was subsequently used for transductional mapping of nine linked chromosomal genes in *B. thuringiensis* [92].

#### 4.2.4. Other Transducing Phages

Other temperate phages (**ϕHD67**, **ϕHD130**, **ϕHD228** and **ϕHD248**) capable to mediate transduction among *B. thuringiensis* sv. *aizawai* strains and heterologous transduction between serovars *aizawai* and *kurstaki*, were isolated after mitomycin C and UV induction of *B. thuringiensis* sv. *aizawai* [93,94,95]. Phage genomes sizes were estimated using restriction analyses as follow: ϕHD67: 45,730 bp; ϕHD130: 38,120 bp; ϕHD228: 36,060 bp and ϕHD248: 47,150 bp [148]. Since phage ϕHD248 contains the largest DNA and should transduce larger fragments of bacterial chromosome, it was therefore used for genetic analysis in *B. thuringiensis* sv. *aizawai*. It was found that ϕHD248 has a broad host range, plating on 7/14 serovars of *B. thuringiensis*, which makes it a good candidate to be used as cloning vector. Its dsDNA genome appears to have a circular permutation and lack cohesive ends. However, cohesive ends might be very unstable and separated even in the absence of disruptive conditions, thus it should be confirmed by a genome sequencing approach. This phage proved to have potential to be used for fine-structure chromosomal mapping, identifying two linkage groups in *B. thuringiensis* sv. *aizawai* [148].

An interesting temperate transducing phage, called **TP21**, has been identified in *B. thuringiensis* sv. *kurstaki* HD-1 [149], and so far, this might be the only specialized transducing phage for the *B. cereus* group [97]. However, since this phage has a “plasmidial” prophage state it will be discussed later (see Section 4.4.1.).

Phage **Tt91** was also isolated from soil and has a broad lytic spectrum that includes several *B. thuringiensis* strains. It was determined that this large phage belongs to the *Myoviridae* and is able to perform intervariant (among different serovars) transduction [80]. Another intervariant transducing phage, **Tg13**, is able to transduce genetic markers between *B. thuringiensis* serovars *galleriae* and *dendrolimus* at a frequency of 10^−7^. This phage has a broad host range and its estimated DNA molecular mass is 40.3 MDa (61 kb) [78]. The temperate phage **Px1** was isolated from the culture of *B. thuringiensis* sv. *galleriae* 69/6 producing enthobacterin [86]. The ultrastructural analysis showed that this siphovirus has an isometric multifaceted head (B1 morphotype) with 40 nm in diameter. The length of its non-contractile transversely lined tail is 130 nm. It is very sensitive to chloroform, a feature shared with tectiviruses (see Section 4.4.2.). The phage is shown to be capable of efficient plasmid transduction between bacteria belonging to *B. cereus* group [86]. Other transducing phages have been reported for *B. anthracis*, *B. cereus* and *B. thuringiensis*, but unfortunately there is little information available about them, and therefore they will be not discussed here. In addition, Sorokin has addressed the potential existence of other transducing phages among the available *B. cereus* group phage genome sequences in an excellent analysis (for a review see [150]).

One interesting characteristic shared by many of the transducing phages for *B. anthracis*, *B. cereus* and *B. thuringiensis*, is that they have been isolated from soil samples. This is not totally surprising, since the soil has been proposed as a reservoir of spores of these closely related bacteria [19,151]. What is however interesting is that the presence of transducing phages opens our view of how these bacteria and phages might communicate and evolve in this ecological niche. An in-depth genome analysis of the *B. cereus* group transducing phages should provide new insights concerning their “natural roles” in soil.

### 4.3. Phages with a Chromosomal Prophage State

With the increasing number of bacterial genomes sequenced, it has become evident that the majority of bacteria contain prophages that substantially contribute not only to the bacterial genetic variability, but also to the evolution of virulence in various pathogens [3,152]. A genetic analysis of prophages integrated into the *B. cereus s.l.* chromosome is beyond the scope of this review. Nevertheless, there are some interesting prophages reported, integrated into *B. anthracis*, *B. cereus* and *B. thuringiensis* chromosomes that will be addressed here. A special subsection is dedicated to the γ-like phages due to their importance in *B. anthracis* identification.

The *B. anthracis* chromosome contains four putative prophages, designated **lambdaBa01** (50,482 bp), **lambdaBa02** (44,043 bp), **lambdaBa03** (16,759 bp) and **lambdaBa04** (37,385 bp) (Table 4), that make up to 2.8% of the total chromosome [16] and facilitate the distinction of this bacterium from other *B. cereus* group species (Table 1). In fact, comparative genomic studies have showed that the four prophage regions represent a high percentage of the unique genes in *B. anthracis* not found in the other *B. cereus* group members [15,16,153,154]. In an extensive study, Sozhamannan and collaborators showed that in more than 300 geographically and temporally divergent *B. anthracis*, the four prophages were conserved, being able to excise from the chromosome at low frequencies (2 × 10^−5^ – 8 × 10^−8^/cell) but appearing to be defective, unable to form viable phage particles or lyse the cells [15]. All four prophages contain genes encoding recombinases and terminal-repeat DNA motifs that may function as attachment (*att*) sites [15]. Moreover, the four prophages do not contain virulence genes and, apart from putative antibiotic resistance determinants and regulatory proteins, the functions of the ORFs are largely unknown. However, several of the prophage genes encode putative membrane or secreted proteins that may play a role in the interaction of *B. anthracis* with external environments such as the mammalian immune system [16].

Other similar prophages are also present in other *B. cereus* group members, but they generally contain genes with little DNA sequence identity to *B. anthracis* prophages genes and are inserted at different chromosomal loci [16]. It has been found that the chromosomes of *B. cereus* E33L, a phylogenetically close isolate to *B. anthracis*, and *B. weihenstephanensis* KBAB4 each contains a prophage homologous to lambdaBa01, but these prophages are inserted in different genomic locations compared to *B. anthracis* [155,156]. Whether these differences provide an evolutionary insight into the relationships between strains harboring lambda01-like prophages still needs to be resolved.

Moreover, three putative prophages have been identified in the genome of *B. cereus* ATCC 10987, whereas the chromosome of *B. cereus* ATCC 14579 contains six putative integrated prophages, mostly uncharacterized [17,155]. Interestingly, the *B. cereus* ATCC 14579 prophage **phBC6A51** (Table 4) genes, which mostly encode proteins with no matches in the databases, are largely up-regulated under swarming conditions [157] (*i.e.*, bacterial collective motility that requires flagella to move over solid surfaces, as evidenced for some members of the *B. cereus* group [158,159]). This fact suggests that the activation of the phBC6A51 genes may provide an advantage to bacteria living in multicellular communities [157]. Also, in some *B. thuringiensis* mutants with attenuated virulence to *Manduca sexta*, the mutation causing the virulence defect was traced back to a disruption of locus 6F8 that codes, among others, for a partial protein that is 45% identical to the large phage minor tail protein of *B. cereus* ATCC 14579 prophage **phBC6A52** (Table 4), a feature that suggests that these prophage-related genes might contribute to insect virulence acquisition in *B. thuringiensis* [160].

**phIS3501** (Table 4) is a siphovirus integrated into the haemolysin II (*hlyII*) gene of *B. thuringiensis* sv. *israelensis* ATCC 35646. The phIS3501 genome has five functional modules: lysogeny and lysogenic regulation, replication, DNA packaging and maturation, and a head and tail structural module and lysis [123]. Phage phIS3501 is able to excise from the bacterial chromosome after induction with mitomycin C. However, it was determined that in the lysis module, the endolysin gene is interrupted by an internal frameshift and consequently, this phage is not able to lyse the host cell [123]. Quite interestingly, excision of phIS3501 from the chromosome results in the restoration of the whole length *hlyII* gene, thus enabling the cells to potentially synthesize the active haemolysin [123].

Recently, Yuan and co-workers found a prophage, named **proCM3** (Table 4), inserted in the chromosome of *B. thuringiensis* strain YM-03 [124]. The draft genome sequencing of this strain pointed out that proCM3 is integrated downstream of an ABC transporter permease-encoding gene with a 55-bp overlap and upstream of another ABC transporter permease-encoding gene. The genome of proCM3 contains genes coding for structural proteins, DNA replication, host lysis, and regulator proteins [124]. Also, proCM3 genome codes for a site-specific recombinase that might be involved in the integration/excision of the prophage genome into/from the chromosome. However, no inducible phage has been detected after mitomycin C treatment [124]. Besides, the genome of this prophage is closely related to the genomes of phages TP21-L (see Section 4.4.1.) and BMBtp2 [124].

The temperate phage **BMBtp2** (Table 4) was induced by mitomycin C treatment from *B. thuringiensis* sv. *tenebrionis* strain YBT-1765 [116]. This prophage belongs to the *Siphoviridae* (B1 morphotype), displaying a typical isometric head (54 nm) and a long non-contractile tail (162 nm). BMBtp2 genome presents the same conserved modular organization found in proCM3, TP21-L and other siphoviruses infecting low-GC-content Gram-positive bacteria [116]. Phage genome comparisons have showed that BMBtp2 has 85% sequence identity to TP21-L [88,116]. Besides, Yuan and collaborators reported that among the 58 predicted ORFs in proCM3 genome, 52 were similar to those of phages BMBtp2 and TP21-L [124], indicating that, most probably, these three phages share a common ancestor.

Another phage, different from proCM3 and named **phiCM3** (Table 4), was also found in strain *B. thuringiensis* strain YM-03 [124]. The genome of this *Siphoviridae* phage is also organized in modules, composed of the late region (genes encoding the structural, host lysis and terminase proteins), the lysogeny-lysis control region (which included the transcription regulator encoding genes) and the early region (which included the DNA replication protein- and integrase-encoding genes). It contains genes that encode a predicted cell division FtsK/SpoIIIE protein and the σ^7^^0^ family sigma factor [124]. The presence of an FtsK/SpoIIIE homolog may suggest a requirement for DNA translocation during the viral infection cycle and might also be involved in regulating sporulation of the host cell. Comparative genome analysis showed that the phiCM3 genome exhibited high similarity with the γ-like phages (see Section γ-like phages). It also shares some DNA identity with phages BtCS33, BceA1 and SpaA1 [124].

Phage **BtCS33** (Table 4) was found in *B. thuringiensis* sv *. kurstaki* strain CS33 [117]. It has a narrow host range and produces small, turbid plaques on sensitive bacteria. This siphovirus has an isometric head (61 × 67 nm) and a long non-contractile tail (204 × 65.7 nm) with tail fibers [117]. As for phiCM3, BtCS33 has a genome structure consisting of three modules and exhibits high similarity in genome organization and amino acid sequences of structural proteins with the γ-like phages. However, they only share 65% amino acid identity in the tail fiber proteins [117], thus possibly explaining they different host-range since the tail fibers proteins have been shown to be essential for the cell wall receptor recognition and binding of γ-like phages [120]. Moreover, the phage-encoded RNA polymerase sigma factor and the FtsK/SpoIIIE protein present in γ-like phages and phiCM3 are also present in the genome of BtCS33 [117].

Furthermore, two lysogenic phages, **MZTP01** and **MZTP02**, were found after induction with mitomycin C in *B. thuringiensis* sv. *kurstaki* strain MZ1, used in commercial fermentations in China [85]. They belong to the *Siphoviridae*, having isometric heads (MZTP01: 75 × 55 nm; MZTP02: 82 × 85 nm) and long rigid tails (MZTP01: 183 × 12 nm; MZTP02: 220 × 18 nm) [85,121]. Based on their host range, stability and antigenicity, these phages appeared to be different [85]. MZTP02 (Table 4) was further characterized and fully-sequenced [121]. Its linear dsDNA is outlined by terminal inverted repeats (40 bp) with terminal proteins linked to the 5’-termini [121], as was shown for tectiviruses (see Section 4.4.2.). These terminal proteins are essential for the replication process [161]. Among the 20 ORFs predicted for MZTP02, six represented unique proteins and nine have similarity to other phage proteins (*i.e.*, two terminase subunits, portal protein, minor head protein, scaffold protein, two putative membrane proteins, tail component, and minor structural protein) [121]. Alignments between MZTP02 tape measure protein (TMP) and various TMPs from known phages showed that lambdaBa01 was the closest relative. The MZTP02 does not contain any identifiable integrase gene, thus it is possible that it does not integrate into the host chromosome and rather exists as linear plasmid, as described for tectiviruses in the *B. cereus* group (see Section 4.4.2.). However, this hypothesis needs to be proven.

Strikingly, while studying a temperate phage named **SpaA1**, isolated from Antarctic soils and infecting *Staphylococcus pasteuri*, Swanson and collaborators found that almost the complete genome (except for the short terminal repeats) from phage MZTP02 was present in one module (region I) of the SpaA1 genome [115]. Moreover, this research group discovered a second phage, **BceA1** (Table 4), in a *B. cereus/B. thuringiensis* strain isolated from Antarctic soils that also includes almost the complete genome of MZTP02 within its own genome [115]. BceA1 is a siphovirus with an isometric head with a diameter of ~63 nm and flexible tails of ~210 nm in length. SpaA1 has the same virion morphology as BceA1, and the genome sequences of both phages are almost identical, except for their ORF47 and the immediate surrounding area [115]. The principal difference between these two phages is related to their host range: BceA1 infects *B. cereus* and *S. pasteuri*, whereas SpaA1 only infects *S. pasteuri* [115]. Additionally, similar inserts to MZTP02 are present in the genomes of *B. thuringiensis* sv. *monterrey* strain BGSC 4AJ1 and *B. cereus* Rock4-2 in the form of a prophage, suggesting that MZTP02 can be shuttled between genomes resulting in chimeric viral genomes [115].

Aside, a temperate phage was isolated after mitomycin C induction of *B. cereus* NCTC 11143, a cereulide-producing (emetic) strain [113]. The phage was named **11143** (Table 4) and was classified as a member of the *Siphoviridae* family by morphology and genome structure. Genome analysis of phage 11143 revealed putative ORFs involved in replication, morphogenesis, DNA packaging, lysogeny, and host lysis [113]. Genomic comparisons at the DNA and protein levels exposed homologous genetic modules with patterns and morphogenesis proteins similar to those of other *Bacillus* phages. Furthermore, phage 11143 genome shares a high similarity with a putative prophage region of the genome of *B. cereus* AH187 (F4810/72) [113], an extensively characterized and reference emetic strain [162], suggesting an evolutionary relationship among these two strains and (pro)-phages.

**Figure 3:**
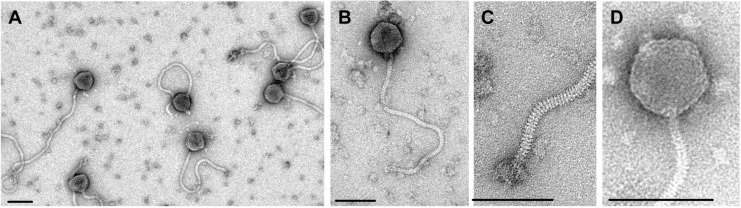
Transmission electron micrographs of phage of phage vB_BanS-Tsamsa particles negatively stained with 2% uranyl acetate on carbon-coated copper grids. (**A**) Preparation overview; (**B**) Close-up of single phage particle; (**C**) Details of the phage tail distal end; (**D**) Details of the phage head structure. Individual capsomers are visible. Scale bars represent 100 nm. Reproduced from Ganz *et al.* (2014), *PLoS One* (reference [125]).

Recently, a novel temperate phage, namely **vB_BanS-Tsamsa** (Table 4), was induced from *B. anthracis* isolated from carcass sites in Etosha National Park, Namibia [125]. Its ~169 kb genome makes it, so far, the largest siphovirus found in *Bacillus*. vB_BanS-Tsamsa displays a long, flexible and non-contractile tail of 440 nm (not including baseplate structure) and an isometric head of 82 nm in diameter (Figure 3). It has individual tail striations (disk-like structures, Figure 3C) and a baseplate with appendages. The head features visible individual capsomers-like structures similar to the ones present in “SPO1-like” phages (Figure 3D) [64,125]. vB_BanS-Tsamsa has a narrow host-range, infecting some members of the *B. cereus* group (*i.e.*, *B. cereus, B. thuringiensis* and *B. anthracis*) and exhibiting moderate specificity for *B. anthraci*s [125]. Phylogenetic analysis using the phage terminase indicated that vB_BanS-Tsamsa clearly differs from previously described phages isolated from *B. anthracis* (*i.e.*, γ-like phages) [125].

Lastly, several other temperate phages have also been studied in *B. thuringiensis* mainly because they accounted for an important reduction—sometimes up to 50%–80%—of the spores and crystal production during fermentations [55,93,94,121,163,164]. However, the available information for these phages is scarce and limited, and therefore they were not included in the present review.

#### The γ-Like Phages

Several phages are known to lyse *B. anthracis* and, among them, the Gamma phage is one of the most extensively characterized. As previously mentioned, Gamma-phage susceptibility is an important diagnostic tool for differentiating *B. anthracis* from closely related *B. cereus* group species (Table 1). Although Gamma phage is highly specific for *B. anthracis*, there are a few *B. cereus* strains that can be infected by this phage [128,165,166]. Therefore, the World Health Organization does not suggest the use of Gamma phage as a sole means for *B. anthracis* identification and detection, but instead it can be used in conjunction with the other tests [23]. Recently, it has been recognized that Gamma is just one representative of a group of closely related phages [118,120], herein referred to as γ-like phages.

The origin of the Gamma phage is somewhat complicated. In 1951, McCloy isolated a phage called **W** from an atypical *B. cereus* strain, namely strain W (ATCC 11950) [100]. It was found that when propagated on *B. anthracis* strain Davis, phage W had a wider range of action than that propagated on strain W, being able to infect all of 171 *B. anthracis* strain tested. At that time, only two of the 54 tested strains of *B. cereus* were susceptible to W. Despite its partial specificity for *B. anthracis*, phage W failed to produce lysis of smooth (encapsulated) *B. anthracis* variants [100]. In a subsequent study, phage W was described as consisting of two forms: phage **Wα** (virulent, rare mutant) and phage **Wβ** (temperate, predominant form) [101]. While the former phage lysed and multiplied on strain W, phage Wβ produced lysis on cultures of *B. anthracis* but did not lyse strain W and smooth variants of *B. anthracis.* Yet, both phage forms were serologically identical [100]. In 1955, Brown and Cherry isolated another virulent variant of phage W, designated **Gamma** (γ) [167]. This phage variant has the unique properties of being highly specific for *B. anthracis*, lysing both smooth and rough (non-encapsulated) strains of *B. anthracis*, but being unable to lyse and propagate on strain W [167]. Since many *B. anthracis* strains are non-encapsulated, Gamma became a valuable typing tool. Nevertheless, during the 1960s phage Wα was used to discriminate among capsulated and non-capsulated variants of *B. anthracis*, as it only lyses the later variants [168]. Besides, it was shown later that *B. cereus* strain W contained a cryptic prophage, denominated ***wx* (**and its virulent form ***wxc***), that is involved in lysogenic conversion to phospholipase A production in *B. cereus* [102,103]. Thus, the presence of these interesting prophages makes *B. cereus* strain W a good example to study today, in order to possible assess the contribution of this type of mobile genetic elements to the different ecotypes and pathotypes present in the *B. cereus* group.

Despite Gamma phage efficacy in identifying *B. anthracis* strains, it was not characterized until mid-1970s [169]. The Gamma virion has an isometric head (52–59 nm in diameter) and a long, non-contractile tail (*Siphoviridae*, B1 morphotype). The semi-rigid tail is 200–217 nm long with a width of 9.5 nm and is connected distally to a small plate and a fibrous tail extension [120,133,169]. Remarkably, Gamma particles generally display a bouquet-like aggregation created by tail fiber adherence to bacterial debris and/or interbase affinity [120]. Its first proteomic characterization indicated that Gamma particles consist of 10 polypeptides, with molecular weights ranging from 12 to 140 kDa, four of which were found to be structural proteins from the isometric head [169]. More recently, a LPXTG-harboring protein, named GamR (Gamma phage receptor), was identified as the bacterial receptor for the Gamma phage [166].

As a result of the widespread use of Gamma phage, there are several γ-like phages that are very similar, yet displaying some genetic differences. The fully-sequenced γ-like phages are (Table 4): the temperate phage Wβ [120] and the virulent phages **Fah**, used widely in the former Soviet Union to identify anthrax bacteria [119]; **Cherry**, originated from Brooks Air Force Base, San Antonio (Texas, TX, USA), though it is not clear if it was isolated at the base [118,170]; **USAMRIID**; **LSU** [118]; isolate **d’Herelle** [120] and **Porton**.

Fah was the first representative to be completely sequenced, revealing that the “left half” of its genome contains genes coding for structural proteins and host lysis functions in an arrangement typical of lambda-like siphovirus. The “right half” of the genome contains genes coding for enzymes of viral genome replication and for many predicted transcription factors that are likely to regulate viral gene expression [119]. These “right half” genes share a high level of sequence similarity and common synteny with a region in *B. cereus* prophage phBC6A51 (mentioned in Section 4.3.). Although Fah forms only clear plaques, its genome encodes an integrase-like protein and several proteins that might mediate lysogenization, including a Cro/CI-like repressor. Yet, it is not clear if Fah’s repressor is functional. Moreover, the presence of 9-nt sequence (5’-CGCCGCCCC-3’) at the junction site between viral DNA ends indicates that Fah genome ends are cohesive and form 3’-extended *cos* site [118,119]. Fah possesses distinct classes of genes that are temporally regulated: early (e.g., transcriptional regulators); delayed (e.g., FtsK/SpoIIIE ATPase and some transcriptional regulators) and late (e.g., structure and lysis) [119]. Fah does not execute host transcription shut-off and depends on host RNA polymerase σ^A^ holoenzyme for transcription of its early and late genes. Additionally, Fah encodes a sigma factor, σ^Fah^, a close relative of *Bacillus* sporulation factor σ^F^ that directs bacterial RNA polymerase to at least one late viral promoter. σ^Fah^ is negatively regulated by host SpoIIAB, an anti-sigma factor that controls sporulation [119].

**Figure 4:**
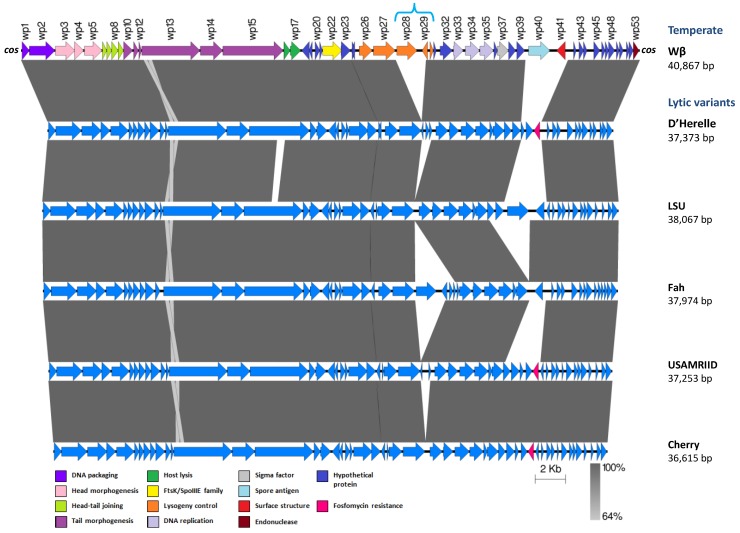
Genome comparisons of γ-like isolates. Predicted genes and direction of transcription are represented as block arrows. For phage Wβ, ORFs are colored according to gene function, as indicated by legend at the bottom. wp indicates numerical gene designations for phage Wβ. Conserved regions are grey-shaded, the color intensity indicating the nucleotide identity levels (from 64% to 100%). The comparisons were done by BLASTn, and similarities with E values lower than 0.001 were plotted. Blue brace above wp28 and wp29 indicates the genes affected by the deletion in the lysogeny control module that characterize the lytic γ-like isolates. When present, Fosfomycin resistant gene is indicated by magenta ORFs (arrows) in the lytic isolates. For further description, see the main text. The figure was produced using Easyfig 2.1 program [171] using data extracted from GenBank annotations and from [118,120]. GenBank accession numbers are listed in Table 4.

Shortly afterwards, two studies that involved the sequencing of Wβ and Gamma (isolate d’Herelle), on one hand [120] and Gamma isolates Cherry, USAMRIID and LSU [118] on the other hand, were published almost in parallel. The first study demonstrated, at a genomic level, that Gamma lytic phage evolved from the temperate Wβ phage in a process involving distinct DNA recombination events and the accumulation of both point and small and large deletions (Figure 4) [120], while the second study confirmed that the genome of the analyzed Gamma isolates, plus Fah, were identical except at three variable loci [118,119].

In particular, genome comparison analysis between γ-like isolates showed that isolates d’Herelle, and USAMRIID arose from Wβ through a 2003 bp deletion in the lysogeny control region (Figure 4), whereas Cherry and LSU arose from identical 2643 bp deletions exactly encompassing the above 2003 bp deletion with an additional 640 bp stretch, removing a Cro repressor homolog and an additional gene [120]. The deletion in the lysogeny control region explains the lytic character of these phages. In addition, a Fosfomycin resistant (Fos^r^) gene (1360 bp) was acquired by each of the lytic γ-like isolates except for Fah and LSU (Figure 4). LSU retains the 2823 bp spore antigen gene present in Wβ [120], but Fah, instead, has a deletion of ~1.1 kb in this region. For isolate d’Herelle it was shown that Fos^r^ gene was functional, but for phages USAMRIID and Cherry it is still not determined if a functional protein is produced. Phage Porton genome was not further analyzed; however, it does not harbor any Fos^r^ gene.

Furthermore, the other two heterogeneous loci, in addition to the Fos^r^ island, are located (*i*) near the phage integrase (wp27 in phage Wβ in Figure 4) and (*ii*) affecting the coding sequence of a putative replisome organizer (wp34 in phage Wβ in Figure 4). By sequence comparisons and PCRs on phage plaques, Fouts and co-workers found that the integrase can exist under three forms (A, B, C) in the γ-like isolates [118]. Phage USAMRIID and Cherry have forms A (3797 bp) and C (1155 bp), while phage LSU possesses forms B (1794 bp) and C, indicating that each phage stock is not genetically pure. The annotated sequence of Gamma phage isolate d’Herelle contains form B, whereas Fah has form A [118]. The second affected locus in the DNA replication region, includes a difference of 13 amino acids, with a total of four different variations observed, with Fah having the highest variability (missing 8 residues out of 13) compared to the other γ-like isolates [118]. Overall, these genetic comparisons permitted to conclude that γ-like isolates are essentially the same phage, containing variations in three distinct locations, and therefore, having a significant heterogeneity within their population [118].

### 4.4. Phages with a Plasmidial Prophage State

In the lysogenic state, most temperate phages physically integrate their prophage DNAs into the chromosome of their bacterial host. However, some prophages are not integrated and their genomes autonomously replicate as circular or linear plasmids in the lysogens [172]. These types of phages are known as phages with a “plasmidial” prophage state (herein referred to as “plasmidial” prophages). In the *B. cereus* group there are some interesting plasmidial prophages that will be described hereunder. It is worth pointing out that the discovery of these prophages has long been overlooked probably because of their nature, *i.e.*, they were believed to be plasmids.

#### 4.4.1. Circular Plasmidial Prophages

In general, tailed phages have a linear dsDNA configuration when they are packed into their virion head. However, upon infection some temperate phages might circularize their genome, instead of integrating into the bacterium chromosome, and exist as circular plasmids in their host. Therefore, they are named “circular plasmidial” prophages. Besides, some phages might integrate into resident plasmids.

As mentioned above, **TP21** is an interesting transducing phage that exists as a plasmidial prophage. The original reference indicates that TP21 was isolated from late exponential cultures of a partially plasmid-cured derivative of *B. thuringiensis* sv. *kurstaki* HD1 (HD1-9) [97,149,173]. It was also shown that this phage is able to transduce some genetic markers at high frequencies (on the order of 10^−7^–10^−4^). However, it was determined that only two markers (*cysC* and *trpB/F*), out of seven markers tested, were transduced into *B. cereus* at higher frequencies than the reversion rates. All the obtained *B. cereus* transductants contained a 44-kb plasmid [173] that was shown later to correspond to phage TP21 [97]. Since transduction frequencies for some markers (*i.e.*, *cys* and *trp*) were higher compared to other markers tested (*i.e.*, *gua*), it was suggested that TP21 mediates specialized transduction. Moreover, it was shown that restriction fragments of transducing phage DNA hybridized to an insertion sequence (IS*231*-like) probe [174], indicating that this region of homology might be a key element for transduction [97]. Nevertheless, the precise mechanism of how TP21 mediates transduction is still poorly understood and, unfortunately, there is no information about TP21 virion morphology. It is also not known why transducing lysates are only obtained from *B. thuringiensis* sv. *kurstaki* HD1-9 and not from its immediate parental strain (HD1-7) or the original *B. thuringiensis* sv. *kurstaki* HD1, which both differ only in the presence of additional plasmids [97,173].

The designation of TP21 has lately been debated. In fact, two other independent phage isolates have been named as TP21. The first one is an isolate that has recently been completely sequenced and re-named **TP21-L** to avoid further confusions with the transducing TP21 phage described above (which in turn was re-named **TP21-T**) [88]. TP21-L belongs to the *Siphoviridae* family (B1 morphotype) with an isometric head of 58.5 nm in diameter and a long, non-contractile, flexile tail of 144.8 nm length and 11.0 nm in diameter. Its genome size is 37.5 kb (Table 4) with fixed invariable ends, displaying the typical modular organization of temperate siphovirus genomes [88]. However, it has not yet been confirmed if TP21-L integrates in the chromosome as a prophage or if it replicates independently as a circular plasmid. TP21-L is genetically very close to phage BMBtp2 (see Section 4.3.). The other TP21 isolate was collected from a Chinese factory in Guangdong province producing *B. thuringiensis* powder [87]. This isolate can be clearly distinguished from TP21-L by its elongated head of 87 × 55 nm and its flexible tail of 140 × 8 nm in size (*Siphoviridae*, B2 morphotype). Therefore, Klumpp and collaborators suggested re-naming this isolate as **TP21-H** [88]. As for TP21-L, there is no evidence that TP21-H is a plasmidial prophage, but both phages were included in this section to clearly make a distinction from the TP21-T transducing plasmidial prophage.

Another representative of a circular plasmidial prophage is phage **ϕ20.** This phage was isolated from *B. anthracis* Sterne 34F_2_ (pXO1^+^ pXO2^−^) after mitomycin C induction and found to have a dsDNA of 48.7 kb. It was shown that ϕ20 genome exists as a plasmidial prophage. ϕ20 has polyhedral heads of 65 nm in diameter and tails of 217 nm long and 15 nm wide, clearly belonging to the *Siphoviridae* (B1 morphotype) [81].

Recently, a phage called **vB_BceS-IEBH** was isolated from the emetic *B. cereus* strain CD555 [126]. Sequencing of this temperate phage indicated that it has a circular genome of 53.1 kb (Table 4). This phage belongs to the *Siphoviridae* family with 55 nm isometric head and a 150 nm long non-contractile tail. It also displays transverse tail disks (5 to 7 per phage particle) that are rarely observed in siphoviruses [126]. However, some siphoviruses displaying transverse tail disks have been isolated from *B. thuringiensis* serovars *tochigiensis*, *yunnanensis*, *shandongiensis*, and *mexicanensis* [164]. Restriction profiles of phage vB_BceS-IEBH DNA indicated that its genome is packaged by a headful mechanism similar to the one of the transducing phage P22 infecting *Salmonella enterica* sv. Typhimurium, in which replicated DNA is “stuffed” into the virion until it is full, rather than filling each virion with a single copy of the sequence [126,175]. In addition, it was shown that phage vB_BceS-IEBH replicates as a circular plasmid in the lysogenic state. Moreover, a 9-kb plasmid-like region composed of 13 ORFs was identified. Inside this region, a fragment of around 2 kb comprising an ORF encoding a putative plasmid replication protein was found and shown to be self-replicating in *B. thuringiensis*. Interestingly, another phage, namely **250**, was isolated from the emetic strain *B. cereus* 250. The phage particles display isometric heads with long non-contractile tails, belonging to the *Siphoviridae* family [114]. The authors do not mention the presence of transverse tail disks similar to the ones exhibited by vB_BceS-IEBH. Notwithstanding this observation, sequence analysis of phage 250 revealed that its circular dsDNA genome (56.6 kb; Table 4 [114]) shares more than 98% DNA identity for more than 80% of the vB_BceS-IEBH genome [126].

**Figure 5:**
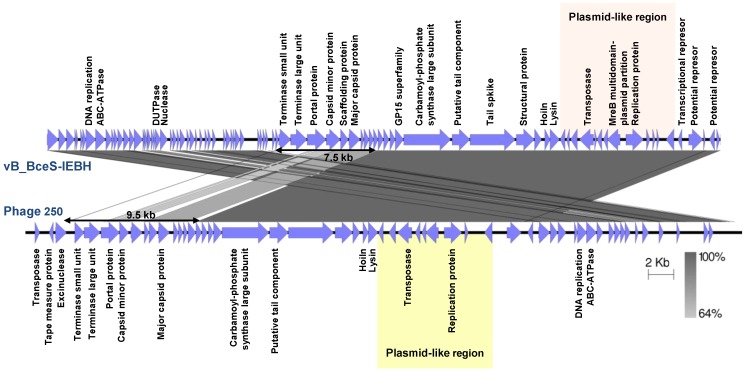
Genome comparisons of phages vB_BceS-IEBH and 250. ORFs are plotted as arrows and some predicted gene functions are indicated. The conserved regions are grey-shaded, the color intensity indicating the identity levels (from 64% to 100%). The 7.5- and 9.5-kb regions with the lowest nucleotide identity are indicated by double-head arrows. The plasmid-like regions for vB_BceS-IEBH and 250 are highlighted in rose and yellow boxes, respectively. Comparisons were done by BLASTn, and similarities with E values lower than 0.001 were plotted. The figure was produced using Easyfig 2.1 program [171]. GenBank accession numbers are indicated in Table 4.

To further illustrate this nucleotide similarity, whole-genome comparisons were done using the Easyfig software that utilizes the BLASTn algorithm [171]. As can be seen in Figure 5, overall there are few small regions of no sequence similarity and some resemblance in genome organization. The largest difference between these two phages is that the 7.5-kb region on the vB_BceS-IEBH genome, containing genes encoding putative terminase and capsid proteins, is replaced by a 9.5-kb fragment in phage 250 that also contains terminase and capsid genes as well as genes with unknown functions [171]. Despite that it has not been determined that phage 250 replicates as a circular plasmid in the prophage state, this striking homology with phage vB_BceS-IEBH (that includes the “plasmid-like” region) suggests that phage 250 might have plasmidial prophage state as well. Further DNA restriction analyses and characterization of the plasmid-like replication region will shed light on the nature of phage 250. Since it has been shown that emetic *B. cereus* strains are restricted to a highly clonal population [162], it is worth noting that vB_BceS-IEBH and phage 250 share only a weak sequence similarity with phage 11143 (see Section 4.3.) [114]. Therefore, the analysis of genomic and plasmidial prophages in emetic *B. cereus* strains might contribute to a better understanding of the population structure of this bacterial lineage.

Few other examples of circular plasmidial prophages in the *B.* cereus group can be found in the literature (e.g., *B. thuringiensis* sv. *israelensis* phage **SU-11** [89]). Nevertheless, the majority of these phages is still poorly characterized. Furthermore, some are not *bona fide* circular plasmidial prophages, but are instead integrated into resident plasmids. For instance, phage **J7W-1** (48 kb) integrates into plasmid pAF101 (69 kb) from *B. thuringiensis* sv. *sotto/dendrolimus* strain AF101. Phage J7W-1 is readily induced by ethidium bromide but not by other commonly used methods such as UV radiation [82]. It can be also induced by temperature or during mating [83,84]. Comparison of restriction patterns of pAFl0l and J7W-1 phage DNA revealed that pAFl0l contains not only the entire phage DNA but also a plasmid-specific DNA region, indicating that J7W-1 genome has been stably integrated into pAFl0l. Phage J7W-1 has a head diameter of 65 nm and a tail length of 290 nm [82], belonging to the *Siphoviridae* (B1 morphotype). Host range tests showed that *B. thuringiensis* serovars *israelensis* and *sotto* have a lytic response to infection by J7W-1. In particular, in *B. thuringiensis* sv. *israelensis* J7W-1 lysogens, a new plasmid was detected, which hybridized with J7W-1 genomic DNA [82]. It is still not clear whether the prophage is integrated into a preexisting *B. thuringiensis* sv. *israelensis* plasmid or whether it forms an independent plasmid. Moreover, it was shown that phage J7W-1 is able to associate with a wider variety of *B. thuringiensis* strains, particularly serovars *aizawai*, *indiana* and *dendrolimus*, for which phage induction was observed in J7W-1 lysogens in the two latter serovars, but not in *B. thuringiensis* sv. *aizawai* [176]. Using a J7W-1 DNA probe, it was found that phage DNA always integrated into the largest plasmid in each of the three *B. thuringiensis* serovars containing different plasmid profiles [176]. In addition, phage-like particles associated with some plasmids have been found in *B. thuringiensis* sv. *israelensis* [177]. Yet, its nature and ecological implications remain to be elucidated.

#### 4.4.2. Tectiviruses

**Figure 6:**
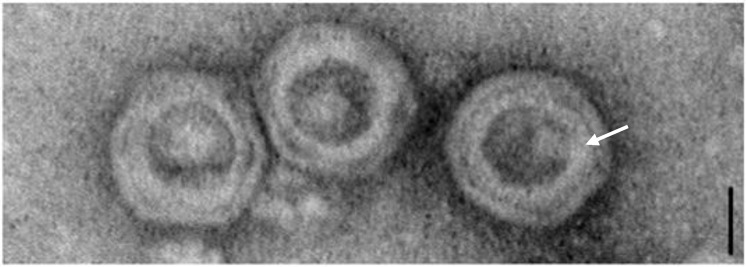
Transmission electron micrograph of phage Wip1 particles negatively stained with 2% uranyl acetate. Phage Wip1 has the typical tail-less isometric heads of *Tectiviridae* with an internal lipid membrane (highlighted by the arrow). Scale bar represents 25 nm. Reproduced from Schuch *et al.* (2009), *PLoS One* (reference [180]).

The family *Tectiviridae* (lat. *tectus* covered) is a relative rare group that includes tail-less phages having a lipid membrane that forms a vesicle beneath the icosahedral protein shell (Figure 6). This membrane is composed of approximately equal amounts of virus-encoded proteins and lipids derived from the host cell plasma membrane [61,178]. The presence of this lipid membrane makes the phage particles very sensitive to organic solvents, mainly chloroform [131]. The relatively rare observation of these phages might be explained by the fact that some traditional protocols for phage isolation include a step applying chloroform. The 15-kb linear dsDNA genomes have long inverted terminal repeat (ITRs) sequences (~100 bp) and are coiled within the lipid membrane. As do Phi29-like phages and adenoviruses, tectiviruses replicate using a protein-primed mechanism that proceeds by strand displacement and can start at both ends of the genome (ITRs contain sites for the replication initiation) [56,179].

Remarkably, this phage family can be subdivided in two distinct groups according to the host they may infect. The well-known PRD1 phage infects Gram-negative enterobacteria such as *Escherichia coli* and *Salmonella enterica* whereas phages **GIL01**, **GIL16**, **Bam35**, **AP50** and **Wip1** (Table 4) are the fully-sequenced representatives of tectiviruses preying on Gram-positive bacteria, specifically on the *B. cereus* group [128,130,131,181,182,183]. Moreover, these two groups of phages have a similar genome size and organization and yet, they have no detectable sequence similarity at nucleotide level [178]. While tectiviruses infecting Gram-negative bacteria are virulent, those present in the *B. cereus* group are temperate phages capable to reside and replicate autonomously as linear plasmids inside the host cell [129,130]. Also, the tectiviruses in Gram-positive bacteria exhibit a strong similarity to the linear plasmid pBClin15 from *B. cereus* ATCC 14579 [129,131], suggesting that this plasmid might be a defective prophage. Tectiviruses have been also reported for *Thermus thermophilus*, but little is known about these phages [184].

In general, the modular genome of tectiviruses in Gram-positive bacteria can be divided in the “plasmid region” that encodes proteins involved in phage genome replication and regulation and ensures the replication of the phage as a plasmid; and the “phage region” that encodes virion structural and DNA packaging proteins and the genes responsible for host recognition and lysis.

AP50 is a tectivirus isolated from soil samples that specifically infects certain strains of *B. anthracis* [128,185]. Likewise, Wip1 was isolated from the intestinal tracts of *Eisenia fetida* earthworms and its narrow host range is highly specific to *B. anthracis* [183]. Wip1 genome is closely related to AP50, but displays some interesting differences in its genome organization, particularly the location of its putative DNA polymerase gene at the 3’ end of the genome (complementary strand) [132], whereas for the other known tectiviruses in Gram-positive bacteria the DNA polymerase gene is located at the 5’ end (positive strand). Recent studies have shown that the host ranges of AP50 and Wip1 are narrower than that of Gamma phage [128,183], the gold standard for identifying *B. anthracis* strains, representing a promising tool for diagnostic. The phages GIL01, GIL16 and Bam35, isolated from *B. thuringiensis*, have the most closely related genomes among the tectiviruses infecting the *B. cereus* group [128,130,131,132,182,186]. Quite interestingly, the host ranges of GIL01 and GIL16 seem to be limited to the *B. cereus* group, except *B. anthracis*, clearly differentiating these phages from AP50 and Wip1 [187].

Despite the fact that the five fully sequenced tectiviruses possess a very well-conserved genome, five novel tectiviruses were discovered lately, namely **Sole**, **Sand**, **Sato**, **Emet** and **Lima**, enhancing the diversity known for these plasmidial prophages [90]. Indeed, a preliminary analysis of their genome, focused on a highly variable region located in the “plasmid region” upstream and downstream LexA (master regulator of the cellular SOS response to DNA damage), showed that overall the five novel members are quite diverse and some of them harbor unique genes with no orthologous in the databases [90]. Sole and Sand were found in *B. cereus* strains isolated from soil from Dubai (United Arab Emirates), whereas Sato and Emet were discovered in emetic *B. cereus* strains. Lima was isolated from *B. mycoides* [90]. Very recently, 47 tectiviruses were also found using genomic variable regions in a worldwide collection of strains belonging to the *B. cereus* group and this study showed that a greater diversity than previously thought exists in tectiviruses infecting Gram-positive bacteria [187]. Further experimental studies will address the intrinsic diversity of tectiviruses and the interactions taken place between these plasmidial prophages and the complex and diverse niches in which they are found.

### 4.5. The Jumbo Phages

Phages designated as jumbo-phages typically exhibit a genome of more than 200 kb and, as a consequence, they have capsids with larger sizes. The jumbo-phages can be seen as derivatives of smaller phages, with the same core genes but with acquired novel genetic functions that have increased its genomes over evolutionary time [188]. One particular characteristic of these phages is that most of the predicted ORFs have no matches in the current sequence databases, and their genomes themselves are so diverse that a detailed comparative analysis is prevented [188,189].

**Figure 7:**
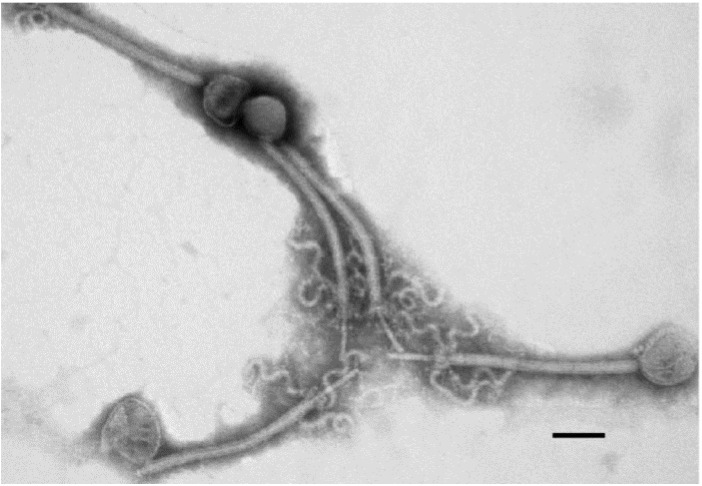
Transmission electron micrograph of phage 0305ϕ8-36. Phage particles are in contact with each other displaying aggregation. The length bar is 0.1 μm; magnification calibration was checked with diffraction grating. The tails of all phages particles have partially contracted. Reproduced from Serwer *et al.* (2007), *Virol. J.* (reference [191]).

Currently, **0305ϕ8-36** is the only *B. cereus* group phage that strictly falls into the jumbo-phage category, with a non-permuted dsDNA genome of 218.9 kb (Table 4), that includes a blunt-ended terminal repeat of 6479 bp [104]. This is an atypical myovirus with a large polyhedral head (95 nm in diameter) and a notable long contractile tail (486 nm in length) (Figure 7). Recently, Pathria and collaborators [190] revised the usual phage purification procedure—ultracentrifugation in a cesium chloride step gradient—to avoid the tail tip-initiated tail sheath contraction and loss of infectivity observed after purification (see reference [190] for method description). This procedure revealed that over 90% of 0305ϕ8-36 particles were multimeric and always joined tail tip-to tail tip [190]. Also, the partial tail contraction observed in Figure 7 is avoided by the revised purification method.

However, the most remarkable feature is that the tail has three large corkscrew shaped fibers (approximately 187 nm long and 10 nm in diameter) that are joined near the baseplate (Figure 7) [192]. Phage 0305ϕ8-36 displays the relatively common genetic organization of myoviruses, including a conserved order of genes within a head structure and morphogenesis module, and a conserved order of modules for head, tail, baseplate and tail fiber proteins. However, it has three novel genes placed on both sides of the head structure module that are thought to be implicated in curly fiber formation [104,193]. On the other hand, phage 0305ϕ8-36 exhibits only limited detectable homology to other phages. The closest homologues for structural or morphogenesis protein-encoding genes and some replication genes are located in a single segment of the chromosome of *B. thuringiensis* sv. *israelensis* ATCC 35646 and in a smaller segment of the chromosome of *B. weihenstephanensis* KBAB4 (phage-like regions BtI1 and BwK1, respectively) [193]. The transcriptional orientation of the majority of predicted ORFs converges on the center of 0305ϕ8-36 genome, dividing it into a left arm and a right arm [193]. Recently, two islands of non-essential genes have been identified in right arm. The first island (3.01% of the genome) includes a DNA translocation operon that begins with a DNA relaxase and continues with a translocase and membrane-binding anchor proteins. The second island (3.71% of the genome) contains genes for two metallo-chaperonins and two tRNAs. Deletion of the first island causes no detectable growth defects, whereas deletion of the second island causes significant growth defects but the phage is still able to propagate [190]. Besides, the non-structural genes of 0305ϕ8-36 include remnants of two replicative systems suggesting that this phage might be originated by fusion of two ancestral viruses [193].

Phage 0305ϕ8-36 was isolated by unconventional phage propagation techniques from soil frequented by cattle in Kingsville, Texas, TX, (USA) using a locally isolated *B. thuringiensis* strain as host [192]. In fact, phage 0305ϕ8-36 does not propagate in the traditional gels used for phage plaque formation and also does not produce visible lysis in liquid cultures. Instead, plaque production and size of plaques highly depend on the concentration of a supporting agarose gel, e.g., 0305ϕ8-36 makes small (<1 mm) plaques in a 0.4% agarose supporting gel, but the size of the plaques become progressively larger as the agarose gel concentration decreases to 0.08% [191,192]. In general, the use of dilute agarose gels represents an efficient method for the isolation of (*i*) large and aggregating phages, (*ii*) phages with long protruding fibers, (*iii*) phages that adsorb to environmental particles and are released when they are in contact with a potential host bacterium, (*iv*) phages that require hydrated polymers for aggressive propagation and (*v*) phages that typically exist in niches with other phages that outgrow them in conventional laboratory culture [190,191,194].

Phage 0305ϕ8-36 forms extensive aggregates (Figure 7) during plaque formation [191]. Plaques are usually clear, except for the border that seems to contain larger phage aggregates than the ones in the clear zone. Clear plaques can also contain roughly circular opaque spots that may resemble phage-resistant host colonies, but instead are aggregates of phage particles [191,195]. The formation of these aggregates seems not to require the presence of either host cells or nucleic acid [195].

The morphological characteristics of 0305ϕ8–36 are almost identical to those of the *B. cereus* phage **Bace-11**, a classified myovirus [70,71]. Phage Bace-11 has an isometric head (92 nm) and a contractile tail of 480 × 20 nm in the extended state with a thick baseplate of 20 × 25 nm displaying also three long curly fibers of 220–230 × 8 nm [70]. Hence, the structure and function of 0305ϕ8-36 and Bace-11 curly fibers are likely to be homologous [104]. Yet, there is no experimental evidence as to what this function might be. Bace-11 was isolated from sewage [69] and, unlike 0305ϕ8-36, it is easily propagated at 30 °C in broth or on agar surfaces by standard methods. However, under these conditions, Bace-11 produces a veiled lysis and poorly visible plaques of 0.1 mm in diameter [70], that might become bigger using diluted agarose gels as for 0305ϕ8-36. The Bace-11 head size suggests that it can package as much DNA as 0305ϕ8-36, indicating that Bace-11 can be another jumbo-phage. Further studies are necessary to shed light on Bace-11 genomic characteristics.

Additionally, the transducing phage TP-13, which has a head size of 120 nm in diameter and a tail of 260 nm in length (referred in Section 4.2.2.), was suggested to have a genome of ~380 kb [79]. With this genome size it would be not only a jumbo-phage, but it will also be considered a girus (contraction of “giant viruses”, whose genomes are >300 kb) [189]. Also, as it was previously mentioned, transducing phages CP-54 and CP-54Ber (referred in Section 4.2.1.) might fall into this last phage category. Advanced genomic characterizations of TP-13, CP-54 and CP-54Ber are needed to determine which category will be appropriate for these phages.

### 4.6. Other Remarkable Phages

As pointed out above, all the sequenced *B. cereus s.l.* phages (as of March 2014) are listed in Table 4. Throughout this review, most of these phages were addressed. However, some phages did not fall in any of the previous sections and, since some of them display remarkable features, they will be shortly described hereunder.

For instance, **vB_BceM_Bc431v3** (Table 4) is a virulent, broad-host-range *B. cereus* myovirus [68]. This phage possesses an isometric head of ~85 nm in diameter with visible individual capsomers and a long contractile tail of ~180 nm in length by ~12 nm in width (Figure 2). The base plate has a cluster of projections that seems to have a central tail fiber. Its morphological characteristics suggest that it might be a “Twort-like” phage. It is able to infect, with different degrees of lysis, strains of *B. cereus*, *B. anthracis*, *B. thuringiensis*, *B. weihenstephanensis*, *Bacillus megaterium*, *Bacillus psychrosaccharolyticus*, *Bacillus licheniformis*, but fails to lyse *B. subtilis*. The phage dsDNA has 20 tRNA genes that deliver 17 different amino acids and does not seem to contain long terminal repeats [68]. vB_BceM_Bc431v3 displays significant sequence similarity, at the protein level, to phages **BCP78** and **BCU4** (Table 4), whose genomes also contain a high number of tRNAs. The acquisition of such a high number of tRNAs might be due to a high virulence nature, since virulent phages replicate faster and need to translate very efficiently their mRNAs [196]. vB_BceM_Bc431v3 genome contains several genes that have rarely been detected in other phages, such as gp143 encoding putative tRNAHis-guanylyltransferase. In addition, it carries some genes that appear to be related to the host sporulation regulators, like a putative segregation protein related to FstK/SpoIIIE DNA transporters, RNA polymerase sigma factor F/B and RNA polymerase sigma factor G [68].

The 162,661 bp genome of *B. thuringiensis* phage **BigBertha** (Table 4) also displays interesting features. It possesses TerL that is homologous to the TerLs of phages with long terminal repeats, having a terminal redundancy of 2577 bp. A total of 287 genes have been identified in its genome, among which 32 are novel hypothetical, 202 are conserved hypothetical, and 53 present BLAST hits that matched known proteins [107]. BigBertha genome contains genes related to DNA and amino acid biosynthesis and/or modification (*i.e.*, S-adenosyl methionine (SAM) methyltransferase, ribonucleotide reductase subunits alpha and beta, dUTPase, dihydrofolate reductase, and thymidylate synthase). It also encodes two sigma factors, three transcriptional regulators and FtsK/SpoIIIE protein [107]. BigBertha has similar organization and homology with *B. cereus* phage **B4** (Table 4), whose endolysin have been proposed to be used as a biological agent to control *B. cereus* propagation (see Section 6.2.) [197]. Also, BigBertha shares some similarity with the genomes of phages **Troll**, **Spock**, and **B5S** (Table 4).

## 5. What about Phages in Other Members of the *B. cereus* Group?

Phages infecting other members of the *B. cereus* group apart from *B. anthracis*, *B. cereus* and *B. thuringiensis*, have been much less characterized. However, as early as 1936, Lewis and Worley evaluated the effect of phages on *B. mycoides* colony polymorphism (dissociation) [198]. Using phages isolated from domestic sewage, they found that *B. mycoides* dissociation occurred much more promptly in the absence of phages than in its presence and that dissociation variants were more susceptible to phage lysis. They also addressed the issue of phages potentially transmitted by sporulating cells [198]. In 1952, Baer and Krueger published a series of three papers studying the effect of lysogeny in *B. mycoides* strain N [199,200,201]. During the decades of 1960–1970, many studies were focused in finding **CAM** phages (referred in Section 3.) [50,51,57], where the majority of *B. mycoides* phages were isolated from soil samples, as for example *B. mycoides* phage No.1 and N17 [202]. There are several CAM and specific *B. mycoides* phages reports focused on their morphologies [51,203], tail 3-D reconstruction [204], inactivation and sensitivity [205,206,207,208,209], antigenic relationship [202], defective morphogenesis [210,211] and phage maturation [212]. However, many of these studies were done in the former USSR, a fact that limits their access. Besides, it is worth pointing out that the lack of information on phages preying on other members of the *B. cereus* group might be due to the fact that species like *B. pseudomycoides, B. weihenstephanensis* and *B. cytotoxicus* are considered as “novel members”, since their taxonomic names were formally accepted only recently, in 1998 and 2013, respectively [20,21,213].

From a genomic point of view, few phages have been reported in other members of this bacterial group. Some prophages have been detected while sequencing the whole genome of particular bacterial strains. For example, the *B. weihenstephanensis* strain KBAB4 has what it seems to be an inducible prophage of 53 kb named **pBWB404** [150,214]. Also, as previously addressed, this *B. weihenstephanensis* strain presents a phage-like region that is partially homologous to the *B. thuringiensis* jumbo-phage 0305ϕ8-36 (referred in Section 4.5.). Moreover, analyses of the chromosome sequence of *B. cytotoxicus* strain NVH391-98 have revealed two regions of ~2690 and ~3010 bp, designated **phBC391A1** and **phBC391A2**, respectively, that contain phage-related genes [215]. This *B. cytotoxicus* strain produces turbid plaques on the closely related strain INRA AF2, but fails to produce plaques on NVH883/00, another close-related strain [215]. Direct sequencing of DNA extracted from plaques produced on strain INRA AF2, indicated that the phage corresponds to phBC391A2 [215]. Whether phBC391A1 is another inducible prophage still needs to be determined.

Lately, the first fully-sequenced virulent phage infecting *B. weihenstephanensis* was described [127]. This phage, named **MG-B1** (Table 4), was isolated from a secondary growth forest soil in Austria using *B. weihenstephanensis* MG01 as host strain. MG-B1 seems to be host specific since it fails to infect 16 other species of *Bacillus* and the type strain *B. weihenstephanensis* DSM11821 [127]. Remarkably, MG-B1 is also the first phage sequenced with podovirus-related genomic features in the *B. cereus* group, being closely related to the Phi29-like group of virulent phages. MG-B1 dsDNA possesses short terminal inverted repeats of 22 nt and, so far, its genome size is the largest (27,190 bp) among members of the Phi29-like phages. Most MG-B1 proteins have similarity with *Bacillus* phages Nf and B103 [127].

## 6. Applications of *B. cereus* Group Phages

Over the past decade, phage research has driven the potential use of phages in modern biotechnology, covering multiple applications, such as bacterial detection and display systems, vaccine development, therapeutic delivery, biological arsenal against multidrug-resistant bacterial infections, natural antimicrobials to prevent food spoilage and to inhibit bacterial food contaminants. Theoretically, *B. cereus* group phages can be used in all these biotechnological applications. However, so far, they have only been tested in specific applications that involved phage typing and biocontrol of bacteria belonging to the *B. cereus* group. Particular examples will be given hereunder.

### 6.1. Phage Typing

The most prominent example for phage typing in the *B. cereus* group is the use of Gamma phage to discriminate *B. anthracis* from the other members of the group, as previously discussed. The *B. anthracis* identification test using the Gamma phage has been validated based on analytic performance parameters accordingly to the USA Pharmacopoeia [216].

Another example is the phage typing scheme developed for *B. cereus* strains isolated from outbreaks or sporadic food poisoning cases [69]. Ahmed and co-workers used 12 phages isolated from sewage (10 *Myoviridae* and 2 *Siphoviridae*) to develop the typing scheme [69]. The results showed that 161 out of 166 *B. cereus* strains isolated from food poisoning cases were typable and indicated a possible link between the etiological agent, the sample source, and the patient in five of six outbreaks situations [69]. Moreover, most of the *B. thuringiensis* strains were also typable using this phage collection that included the jumbo-phage Bace-11 (see Section 4.5.) [69,70].

### 6.2. Biocontrol

Phages represent a promising tool for biocontrolling *B. cereus s.l.* bacteria. They do not only have a great potential for treating bacterial infections (phage therapy), but also to enhance microbiological safety, particularly in the food industry. Phages are suitable to prevent or reduce bacterial colonization in livestock (another type of phage therapy [217]), to decontaminate carcasses and other raw products, to disinfect equipment and contact surfaces and to extend the shelf life of perishable manufactured foods (natural preservatives) [218]. Therefore, some research focused on isolating phages that could provide an additional tool to tackle problems associated with *B. cereus* contamination in food has been done. For instance, Lee and co-workers isolated and characterized two myoviruses, **FWLBc1** and **FWLBc2**, that were able to reduce the concentration of *B. cereus* in mashed potatoes by >6 log_10_ CFU mL^−1^ within 24 h at room temperature, when applied at high concentration [76]. Since these two phages have a relative narrow host range (some *B. cereus* strains), they were proposed to be used as part of a “phage cocktail” [76]. Also, the prevalence of *B. cereus* phages in Korean fermented foods has been studied, finding that around 40% of the samples contained more than one kind of *B. cereus* phage [77]. Besides, one myovirus, **JBP901**, isolated from “cheonggukjang”, a fast-fermented soybean product, has been further analyzed due to its broad host range among *B. cereus* strains. This phage can reduce *B. cereus* growth in liquid culture and in cheonggukjang food, without affecting the growth of *B. subtilis*, necessary for cheonggukjang fermentation [77]. Two additional phages isolated from Korean fermented food products, namely **BCP1-1** and **BCP8-2**, showed that they are also able to control *B. cereus* strains in cheonggukjang, but only if divalent cations were added to the medium [72]. Taken together, these findings show that naturally-food-occurring *B. cereus* phages can readily control food contamination caused by this bacterium.

Another biocontrol strategy, that not necessarily involves the phages themselves, is to use recombinant-phage encoded peptidoglycan hydrolyzing enzymes known as endolysins. These enzymes target and digest the integrity of the cell wall, and are designed to attack one of the five major bonds in the peptidoglycan [219]. After the 2001 anthrax attacks in the USA, *B. anthracis* research aimed to find a specific tool that could have a rapid lethal action against this bacterium and its spores. PlyG lysin, isolated from the Gamma phage binds and kills *B. anthracis* isolates and other members of the *B. anthracis* “cluster” *in vitro* and *in vivo.* Moreover, both vegetative cells and germinating spores are susceptible to PlyG [165]. Likewise, PlyB, identified from the **BcpI** phage, exerts a potent lytic effect on the *B. anthracis*-like strain ATCC 4342 [99]. Expanding the same approach, endolysins have been explored to be used against food-borne pathogenic strains of *B. cereus*. For example, LysBPS13, encoded by the genome of phage BPS13 (Table 4) showed antimicrobial activity against *Bacillus* species and some Gram-negative bacteria when treated with EDTA. The strongest antimicrobial activity was against *B. cereus* ATCC 10876 [108]. LysBPS13 has a remarkably high thermostability in the presence of glycerol and retains its lytic activity after incubation at 100 °C for 30 min, features that make it an excellent candidate to be employed in the industry to control *B. cereus* [108]. Also, the endolysin from *B. cereus* phage B4 (Table 4), designated LysB4 (L-alanoyl-D-glutamate endopeptidase), showed an interestingly broad activity spectrum, lysing bacteria as diverse as *B. cereus*, *B. subtilis*, *Listeria monocytogenes* and some Gram-negative bacteria treated with EDTA [197]. PlyBa, Ply21 and Ply12 are endolysins proteins from *B. cereus* phages **Bastille** (Table 4), TP21-L (Table 4; referred in Section 4.4.1.) and **12826**, respectively, that have been produced as recombinant proteins and showed a rapid and specific lysis of viable cells of several *Bacillus* species, with highest activity on *B. cereus* and *B. thuringiensis* [98]. Also, the two encoded endolysins from GIL01 (Table 4; referred in Section 4.4.2.), Mur1 and Mur2, have been shown to degrade cell wall preparations [220]. While Mur2 degrading activity is limited to *B. thuringiensis* sv. *israelensis*, Mur1 has a broader spectrum, including *B. subtilis* and *Micrococcus lysodeikticus* cell walls [220]. The previous endolysin examples represent good candidates as alternative antibacterials for the treatment, prophylaxis and biocontrol of bacteria belonging to the *B. cereus* group.

As a final commentary, it is important to consider that even though most phages do not, *a priori*, display any risk for human health or the environment, they are dynamic entities that interact with their bacterial host. As a result, in some circumstances, they might raise some biosafety concerns by bringing and potentially disseminating new genetic traits among bacterial populations that can negatively impact the human and animal health [221]. Therefore, a rigorous risk assessment is necessary prior to using *B. cereus* group phages and phage-derivative products. In general, the selection of a specific phage for biocontrol applications requires fulfilling a number of characteristics, such as: be strictly virulent, display a broad host range, be unable to perform generalized transduction, and not perform lysogenic conversion of its host [222]. Also, detailed molecular characterization of the phage genome is mandatory to exclude the presence of any toxin genes or antibiotic-resistance genes [223]. In addition, if *B. cereus* group phages are going to be used as therapeutic agents, they are regulated in Europe by the current framework related to medicinal products for human use (European directive 2001/83/EG) (for further information about phage legal issues and risk assessment, see [223] and [221], respectively).

## 7. Concluding Remarks

Whereas intensive work has been done in the *B. cereus* group to decipher the contribution of plasmids, especially those directly involved in pathogenicity, less attention has been paid to understand the contribution of phages to the adaptation of this bacterial lineage into their different environmental niches. Rapidly after their discovery, *B. cereus* group phages became important tools for genetic and genomic studies thanks to their transducing properties, feature that propelled interest during their successful period of the decade of the 1970. Afterwards, the emergence of other genomic tools, like cloning and sequencing, overshadowed the importance of this type of phages. A renascence of interest on *B. cereus* group phages occurred during the late 1990s, when many representatives of different phage groups were found. Besides the transducing phages, others with particular lifestyles and/or lysogenic states have been described lately, such as phages with chromosomal or plasmidial prophage states, jumbo-phages and γ-like phages. Currently, it is recognized that the gene pool of phages that infect the *B. cereus* group, in particular prophages, is larger and more diverse than that of the rest of the bacterial chromosome. New genomic data are clarifying our understanding of the structure, distribution and variability of these phages. However, several factors need to be considered and further studied to understand the influence of phages in the evolution of the *B. cereus* group lineage. In conclusion, phages preying on this bacterial group do not only have an important contribution to the bacterial genomic pool, but also offer a versatile toolbox with promising biotechnological applications. With the renewed interest in the *B. cereus* group phages and the highly efficient, rapid, and low cost DNA sequencing platforms available today, it is expected that a large number of phages will be discovered and described in the near future.
